# Modeling and Measurement of an Ultrasound Power Delivery System for Charging Implantable Devices Using an AlN-Based pMUT as Receiver

**DOI:** 10.3390/mi13122127

**Published:** 2022-12-01

**Authors:** Antonino Proto, Libor Rufer, Skandar Basrour, Marek Penhaker

**Affiliations:** 1 Department of Neuroscience and Rehabilitation, University of Ferrara, Via Luigi Borsari, 46, 44121 Ferrara, Italy; 2 Department of Cybernetics and Biomedical Engineering, VŠB—Technical University of Ostrava, 17.listopadu 2172/15, Poruba, 708 00 Ostrava, Czech Republic; 3 Université Grenoble Alpes, CNRS, Grenoble INP, TIMA, 38000 Grenoble, France

**Keywords:** ultrasound, acoustic, energy transfer, Langevin transducer, pMUT, lumped parameters model, implantable medical devices

## Abstract

Ultrasound power delivery can be considered a convenient technique for charging implantable medical devices. In this work, an intra-body system has been modeled to characterize the phenomenon of ultrasound power transmission. The proposed system comprises a Langevin transducer as transmitter and an AlN-based square piezoelectric micro-machined ultrasonic transducer as receiver. The medium layers, in which elastic waves propagate, were made by polydimethylsiloxane to mimic human tissue and stainless steel to replace the case of the implantable device. To characterize the behavior of the transducers, measurements of impedance and phase, velocity and displacement, and acoustic pressure field were carried out in the experimental activity. Then, voltage and power output were measured to analyze the performance of the ultrasound power delivery system. For a root mean square voltage input of approximately 35 V, the power density resulted in 21.6 µW cm^−2^. Such a result corresponds to the data obtained with simulation through a one-dimensional lumped parameter transmission line model. The methodology proposed to develop the ultrasound power delivery (UPD) system, as well as the use of non-toxic materials for the fabrication of the intra-body elements, are a valid design approach to raise awareness of using wireless power transfer techniques for charging implantable devices.

## 1. Introduction

In the current era of the Internet of Medical Things, wireless energy transfer can be a solution for charging low-power devices, especially where supplying energy through wires is difficult or inappropriate. This is the case with implantable medical devices (IMDs), which are placed inside the body and whose lifetime is limited by the operating time of battery [1,2]. IMDs, such as chronic pain neurostimulators or combinations of pacemakers and defibrillators need battery replacement every 5 to 10 years, which is costly and risky because the necessary surgery may introduce infections [3,4]. Moreover, with the increased use of implantable smart technologies to regulate organ functions and control prostheses, the IMDs require more and more energy to interface the peripheral and central nervous systems [5].

The IMD lifetime can be increased by harvesting the energy from biological sources, such as thermal gradients, vibration within the body, or biofuel cells. However, these energy sources do not produce enough electricity for most of IMDs, and body tolerance to undesired chemical or biological reactions remains the real challenge to address [6,7].

Nowadays, research efforts have focused on the wireless energy transfer to charge IMDs where an external link delivers energy in the form of electromagnetic radiation or acoustic waves [8]. Acoustic waves for medical applications are generally delivered at ultrasonic frequencies in the form of ultrasound. In comparison with electromagnetic radiations, ultrasound can propagate through electrically conductive materials without being affected by electromagnetic fields. Moreover, ultrasound allows a higher power intensity threshold for safe operation. For diagnostic medical imaging, the U.S. Food and Drug Administration (FDA) sets the ultrasound intensity parameter of spatial-peak temporal-average (I_SPTA_) equal to 720, 430, 94, 17 mW cm^−2^ for peripheral vessel, cardiac, fetal, and ophthalmic applications, respectively [9]. These values are about two orders of magnitude higher than limits set by the U.S. Federal Communications Commission and IEEE for radio frequency (RF) exposure, which are approximately in the range 1–10 mW cm^−2^ [10,11]. A further advantage of ultrasound is less attenuation in tissues; in fact, the attenuation coefficient for 1 MHz ultrasound is 0.6 dB cm^−1^, compared to 9.2 dB cm^−1^ for 100 MHz RF [8]. Ultrasound techniques are also widely used for domestic and industrial needs, for example, for object localization and tracking systems, cleaning solutions, air and water navigation, and in speech processing applications, among others [12,13,14].

Awareness in the use of ultrasound power delivery (UPD) systems for charging IMDs was achieved about 10 years ago by using centimeter-scale transducers. In 2010, Ozeri and Shmilovitz [15] proposed the most effective UPD system ever seen before. They fabricated a disc-shaped piezoelectric plate of 1.5 cm diameter and 0.3 cm thickness, operating in continuous wave mode at 673 kHz. For a given transmitter (TX) to receiver (RX) distance of 5 mm, the UPD system was able to generate a load power of 70 mW for a power transfer efficiency of 27%. However, the efficiency decreases as the TX-RX distance increases. In a study proposed by Mazzilli et al. in 2014 [16], they measured a system efficiency of 1.6% while transferring ultrasound power at 10.5 cm TX-RX distance by using an RX with cross-section area of 0.5 cm^2^. About commercialized centimeter scale device for UPD, Piezo Energy Technologies LLC company, Arizona, developed a portable system able of accomplishing it. It was the research effort of Radziemski and Makin, who together demonstrated continuous stable power transfer at a steady current of 70 mA by means of circular piezoelectric transducers of 2.5 cm diameter for both TX and RX structures [17].

Nowadays, the idea of having a UPD system, including a wearable TX that supplies energy to a network of IMDs within human tissues, is a challenge for many research groups. In this scenario, the most important technological challenge is to reduce the device size [18,19].

By reducing the piezoelectric plate dimension to the millimeter scale, the operating frequency shifts to higher values. For a piezoelectric plate working in thickness mode, the plate thickness of about 1.5 mm results in an operating frequency between 1 and 2 MHz [20]. Working at frequencies above 1 MHz leads to higher wave attenuation, which may result in heating of tissues if the exposure time is prolonged [21]. The maximum temperature increase, in situ, would not exceed 2 °C. To avoid this issue, intermittent ultrasound waves can be delivered from TX to RX with a set duty cycle to control the rise of temperature in situ. Such miniaturized piezoelectric plates are widely used in implantable brain devices for wireless optogenetic stimulation [22]. Another issue of setting an operating frequency above 1 MHz regards the impedance matching problem. Usually, coupling materials and circuits for adaptive matching are used to avoid it [23].

In order to maintain reduced size for the RX while working below 1 MHz, piezoelectric diaphragm structures are an innovative solution [21]. Compared to a piezoelectric plate working in the thickness “33” mode, the piezoelectric diaphragm works in the bending “31” mode and is composed of less piezoelectric material, resulting in lower energy generation than a plate structure when excited from outside. Nonetheless, for a given geometrical dimension of the piezo material, diaphragm structures can operate at much lower frequencies than plates, so reducing the heating of tissue and having less attenuation of signal within the body [21].

Piezoelectric micromachined ultrasonic transducers (pMUTs), developed with MEMS technology, are widely used to detect and generate ultrasound waves. Thanks to the full maturation of lithography process, pMUTs can have multiple geometries, thus adapting their operation to the needs of the application [24,25]. The piezoelectric diaphragm is usually deposited on silicon substrate which forms a backed air cavity. The pMUT structures can be square- or circular-shaped [26,27], can have one or two electrical ports [28], and they can be linked together to form an array of multiple units or concentric geometries [25,29]. They are implemented in haptic feedback and for gesture recognition, and they can be used as air-coupled actuators and for range finding in in-air applications [30,31,32,33,34].

The most promising work using a pMUT to charge an IMD was shown by Basaeri et al. in 2019 [35]. They evaluate the ratio between the thickness of the piezoelectric diaphragm to the thickness of the silicon substrate to obtain the maximum power output. For a 2 mm × 2 mm square diaphragm with a silicon substrate of 50 µm, they found an optimum piezoelectric thickness of 20 µm. To verify their numeric simulations, the pMUT was tested in water at 2 cm distance from the TX. For a given power intensity input of 322 mW cm^−2^, the pMUT delivered an average power, to a pure resistive load of 4.3 kΩ, of about 0.7 mW at an operating frequency of 88 kHz, which is a value much lower than the operating frequency for a piezoelectric plate. The advantage of working at lower operating frequencies is an improvement related to the impedance matching between the piezoelectric transducers and the human body tissue. If the operating frequency is less than approximately 70 kHz, no coupling material is needed to match the impedances [21]. In the work of Basaeri et al. [35], the piezoelectric material used as the active element for developing the pMUT is lead zirconate titanate (PZT). The PZT material is toxic and can lead to the body’s immune rejection, thus becoming an obstacle to the widespread clinical application of IMD for personalized medicine [36].

In this work, aluminum nitride (AlN) material was used to develop a pMUT for testing in a UPD system. The non-toxicity of the AlN increases the sensor biocompatibility and reduces the tissue mismatching for a longer functionality. The proposed AlN-based pMUT is tested in this work to add valuable information on the research topic of wireless energy transfer for charging IMD without having surgery. The modeling of the entire UPD system is therefore given to optimize the amount of power on the RX side.

## 2. Materials and Methods

### 2.1. The UPD System Model

**Figure 1:**
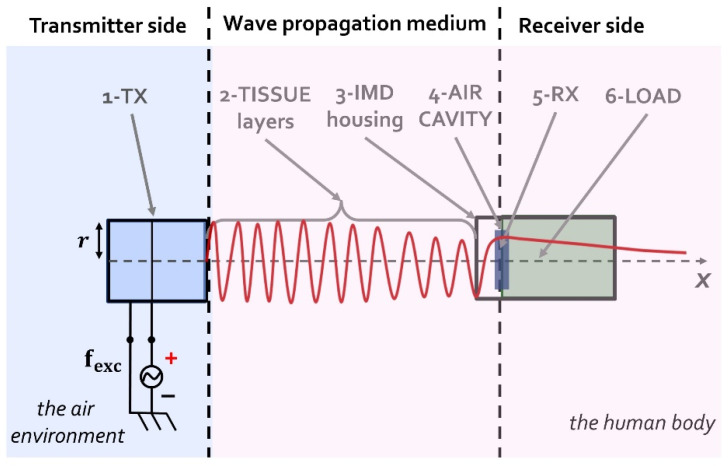
The six blocks representing the UPD system.

Figure 1 shows the six blocks representing the UPD system. Block (1) is the piezoelectric plate transmitting the ultrasound. Block (2) represents the tissue layers in between the TX and RX structures. The tissue layer can be represented by solely the skin, but also with a combination of skin, muscles, and body fat. Block (3) is a thin layer of metal that depicts the IMD housing. Common materials for hermetically sealing implants are titanium or stainless steel (SST). Block (4) is an air cavity. Into the air cavity the RX is placed, and it resonates based on the resulting acoustic pressure within the coupling cavity. Block (5) is the piezoelectric diaphragm structure in the form of pMUT, and block (6) represents schematically the RX electrical load. In terms of the energy transfer, the load is a pure resistive component representing the input impedance of the power management circuitry of the IMD.

Some geometric relations have been taken into consideration to optimize the performance of the UPD system. Firstly, the piezoelectric RX should be placed at a distance equal or greater than the Rayleigh distance (RD) in order to avoid the near-field region. It is known from acoustics theory that a mechanical wave generated by a source converges to a natural focus at the transition between near- and far-field regions, where it assumes a stable value. In the near field, the amplitude of the generated ultrasound wave at a given point is difficult to predict as it oscillates between two extremes and can vary with small changes in location. Calculation of the RD distance is given by:

(1) $$\mathrm{RD}=\frac{r_{\mathrm{TX}}^{2}}{c_{\mathrm{medium}}}\;\cdot \;f_{exc}$$

where $r_{\mathrm{TX}}$ is the TX radius, $c_{\mathrm{medium}}$ is the value of sound speed into the propagating medium, and $f_{exc}$ is the excitation frequency given by an external electrical generator.

A further geometrical consideration for the design of the UPD system relates to the value of the air cavity thickness, $t_{cav}$, which should be less than a quarter of wavelength in order to avoid the generation of standing waves within the coupling cavity. Thus, to calculate the maximum value of $t_{cav}$, to comply the above-mentioned geometrical consideration, the following equation is computed:

(2) $$t_{cav}=\frac{1}{4}\;\cdot \;\lambda_{cav}=\frac{1}{4}\;\cdot \;\frac{c_{air}}{f_{exc}}$$

Regarding the IMD housing, its thickness usually varies between 100 and 400 µm. To conclude, in order to obtain the maximum amount of power transfer in the UPD system, the value of the resonant frequency of the RX should correspond to the value of the resonant frequency of the TX.

### 2.2. Elements Constituting the UPD System

**Figure 2:**
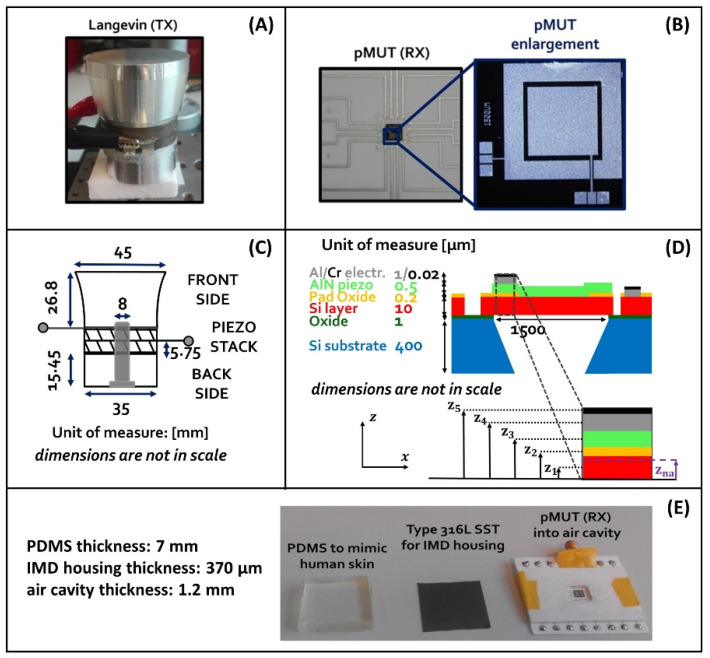
Elements constituting the UPD system. Langevin transducer (**A**); square pMUT (**B**); Langevin transducer geometrical dimensions (**C**); square pMUT structure (**D**); medium layers and air cavity structure for the pMUT placement (**E**).

The following, Figure 2, shows the elements constituting the UPD system in the proposed study.

The TX is a Langevin transducer made by a stack of piezoelectric plates comprised of a solid waveguide on the front side and the backing on the other side. In this case, two outward oriented plates of SM118 piezoceramic material are linked together with a steel screw bolt, which ensures the mechanical pre-stress and the electrical parallel connection of the plate electrodes. The backing side is made of steel and the front waveguide is made of aluminum.

The RX is a uniform square pMUT with lateral side of 1.5 mm. It is a laminate structure formed on a silicon (Si) substrate by a silicon oxide (SiO_2_) thin layer, a nontoxic AlN piezoelectric diaphragm covered by Al/Cr metal pads and backed by an air cavity of cross-section area, $A_{m}$, which can be approximated to a third of the AlN diaphragm cross-section area, S [37]. Electrodes of the pMUT are placed at the edges of the diaphragm above the AlN film. The top electrode is an Al/Cr metal pad, while the bottom is formed by a doped silicon layer. The square pMUT (RX) is fabricated using the PiezoMUMPs^TM^ process flow [38].

Table 1 shows the values of parameters characterizing the piezoelectric material in both TX and RX.

Table 2 shows the values of parameters of all layers constituting the pMUT.

Regarding the medium layers, polydimethylsiloxane (PDMS) material—Sylgard^®^ 184—was chosen to mimic the human skin because of its density and speed of sound values, which are like those of the body. The PDMS is formed by a base part and a curing agent, which were mixed at a ratio of 10:1. Then, the resulting compound was poured into a mold and later placed into a furnace at 70 °C for 2 h. The housing of the IMD is a thin layer of Type 316L SST. It is a bio-compatible material used for designing implants.

### 2.3. Measurement Setup

For the characterization of the TX and RX, measurement of their impedance and phase values were carried out by means of the impedance analyzer IM3570—HIOKI, Hioki Europe Gmbh, Eschborn, Germany. Moreover, for the TX, velocity and displacement values were obtained using together a Polytec OFV-502 Fiber Optic Interferometer and a Polytec OFV-3001 Vibrometer Controller, Polytec, Baden-Württemberg, Germany.

A LabView 2019 software (SW) application, National Instruments, Austin, Texas, United States, to sweep the excitation frequency in a defined value range, was used to control the amplitude of the input signal generated by an Agilent 33500B waveform generator, Keysight Technologies, Santa Rosa, California, USA. The signal generated by the waveform generator goes through a high voltage amplifier, WMA-300 Falco System, Falco Systems, Amsterdam, Netherlands, which is used to amplify the input signal and to adapt, as much as possible, the capacitive behavior of the TX. Then, the output signal of the Falco System is the electrical input signal of the TX.

Acoustic pressure measurements were carried out through a calibrated 1/8-inch pressure-field microphone, Brüel & Kjær Type 4138, connected to a conditioning amplifier, Brüel & Kjær Type WH-3219, Brüel & Kjær, Nærum, Denmark. The microphone was placed perpendicular to the TX surface at the center point of the radiating area.

All the measurements carried out in this study were displayed on an Agilent DSO-X 2002A digital oscilloscope, Keysight Technologies, Santa Rosa, California, USA, and in the LabView 2019 SW application, National Instruments, Austin, Texas, USA. The results were stored as .csv file for post-processing in MATLAB^®^ SW environment.

**Figure 3:**
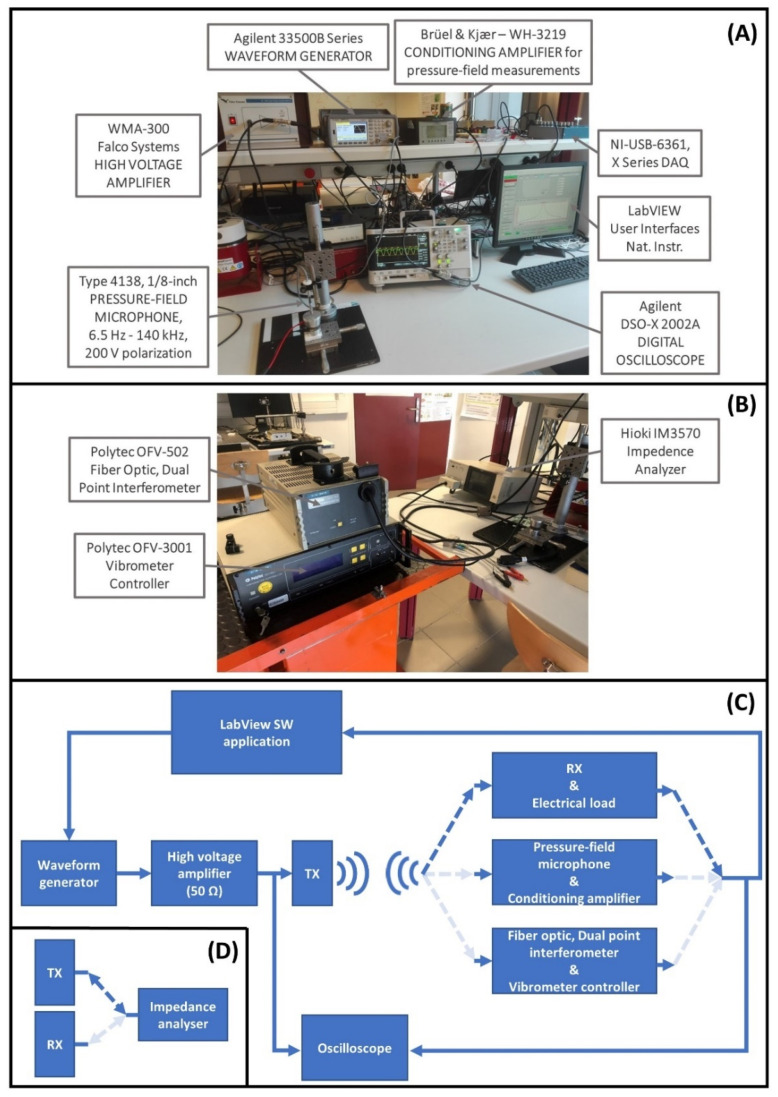
Experimental bench. Waveform generator, high voltage amplifier, conditioning amplifier for pressure-field microphone output, oscilloscope, and National Instrument DAQ and LabVIEW (**A**). Impedance analyzer and Polytec Vibrometer controller for dual point interferometer (**B**). Schematic block diagram of the circuit used for measuring the electrical power output on the load, the acoustic pressure field, and velocity and displacement values for the TX (**C**). Schematic block diagram to measure impedance and phase values of TX and RX (**D**).

Figure 3 shows the experimental bench including all the instrumentation used to carry out the characterization of the TX and RX as well as the measurements of acoustic pressure field and the values of voltage and power output.

## 3. Results

The results section provides data about the characterization of the TX and RX as well as the results of the measurements for testing the proposed UPD system.

The characterization of the TX and RX regards measurements of the values of impedance, phase, velocity, and displacement for the TX and measurements of the values of impedance and phase for the RX. Moreover, measurements to show the acoustic pressure field in air of both the TX and RX were carried out before to test the performance of the proposed UPD system.

While testing the UPD system, the results are given in terms of root mean square (RMS) values of voltage and power output over a resistive load.

### 3.1. Measurements for the TX and RX Characterization

**Figure 4:**
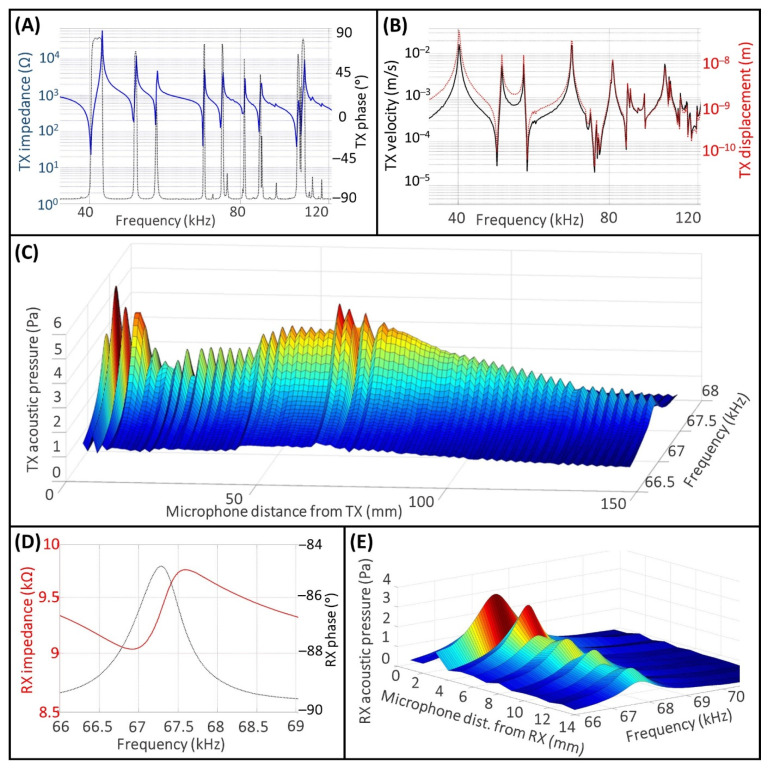
Characterization of the TX and the RX. Impedance and phase of TX (**A**); velocity and displacement of TX (**B**); TX acoustic pressure in air (**C**); impedance and phase of RX (**D**); RX acoustic pressure in air (**E**).

The results of the experimental activity for the characterization of the TX and the RX are shown in Figure 4.

In Figure 4A,B, the values of impedance, phase, velocity, and displacement of the TX are displayed on the frequency range between 20 and 140 kHz. Although the TX is designed to operate at 40 kHz [39], it exhibits also higher mode resonances, which are clearly visible in Figure 4A. This behavior of the TX is due to the complex system composed by the piezo stack and the loads on the back and front sides.

Regarding the operating frequency of interest for the proposed experiment, $f_{\mathrm{exc}}$, which corresponds to a resonant frequency of the TX, $f_{r}^{\mathrm{TX}}$, it must be as closely as possible to the value of the resonant frequency of the receiver, $f_{r}^{\mathrm{RX}}$, in order to optimize the amount of power transfer for the UPD system [21]. Based on this, the selected operating frequency value, $f_{r}^{\mathrm{TX}}$ = $f_{\mathrm{exc}}$, is 67.34 kHz. Indeed, this value is comparable to the value of $f_{r}^{\mathrm{RX}}$ shown in Figure 4D, which was measured with the impedance analyzer and found to be 66.92 kHz.

Regarding the impedance value of TX, $Z_{\mathrm{TX}}$, measured to $f_{r}^{\mathrm{TX}}$, is approximately 50 Ω, the velocity, $v_{\mathrm{TX}}$, is 0.016 m s^−1^, and the displacement, $x_{\mathrm{TX}}$, is 38 nm. Regarding the RX, the impedance value, $Z_{\mathrm{RX}}$, measured to $f_{r}^{\mathrm{RX}}$, is approximately 9.04 kΩ.

In Figure 4C we measured the acoustic pressure field in air for the TX on the frequency range in the surrounding of $f_{r}^{\mathrm{TX}}$, from 66.7 to 67.7 kHz. The peak-to-peak amplitude of the input voltage signal was set to 2.5 V, and the maximum root mean square (RMS) value of the acoustic pressure field to the $f_{r}^{\mathrm{TX}}$, $p_{\mathrm{rms}}^{\mathrm{TX}}$, was approximately 6 Pa in the near field close to the TX emitting surface, and 5 Pa at the Rayleigh distance. Regarding the acoustic pressure field generated in air by the pMUT, Figure 4E, the maximum obtained $p_{\mathrm{rms}}^{\mathrm{RX}}$ value is approximately 3 Pa when the distance between the microphone and the pMUT is 2 mm, and the $p_{\mathrm{rms}}^{\mathrm{RX}}$ value is around 1 Pa at a distance of 14 mm.

### 3.2. UPD System Measurements

**Figure 5:**
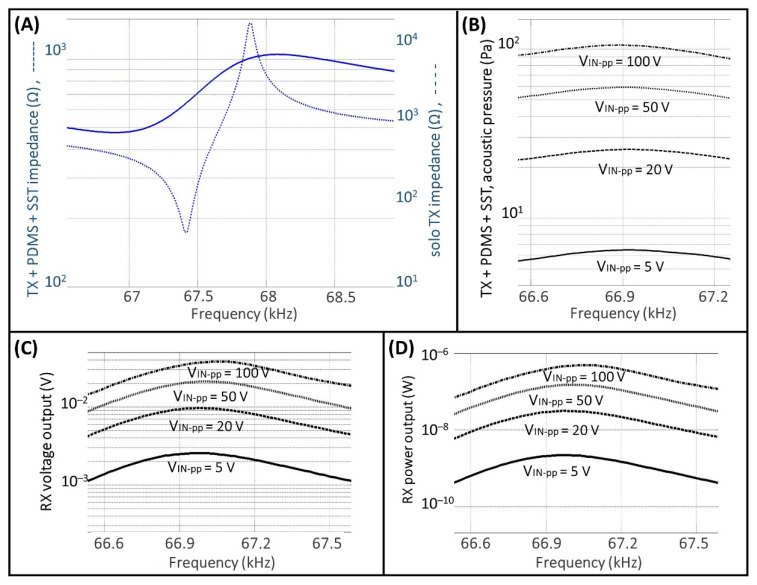
UPD system measurements. Comparison between the impedance value for the free TX and for the TX covered by the PDMS and the SST layer (**A**); acoustic pressure field (**B**); RX voltage output (**C**); RX power output (**D**).

Figure 5 shows the results of measurements carried out for testing the UPD system.

To define the geometry properties of the UPD system investigated here, the thickness of the SST housing layer was calculated using Equation (2), while the RD distance (34.8 mm) was computed using Equation (1) where the value of $c_{\mathrm{medium}}$ was the value of the speed of sound for the PDMS material. The value of the thickness for the PDMS was equal to 7 mm because we considered 26.8 mm for the thickness of the aluminum front side and 1.2 mm for the thickness of the air cavity.

Figure 5A shows the comparison between the impedance value for the free TX and for the TX covered by the PDMS, and the SST layers, which is the actual configuration used in the UPD system. While the TX is covered by the layers of PDMS and SST, the resonant frequency $f_{r}^{\mathrm{TX}}$ is shifted to the left by 66.9 kHz, and the impedance value is approximately 475 Ω. The narrow resonant peak for the free TX is damped when the TX is covered with the PDMS and SST layers, and the useful frequency range becomes reasonably wide to cover the optimal frequency range of the pMUT.

Figure 5B shows the results of measurements regarding the RMS values of the intensity of the acoustic pressure field over the housing of the IMD, when the TX is driven near the resonance with different values for the input peak-to-peak voltage ranging from 5 to 100 V. These values represent the acoustic pressure intensity into the air cavity where the pMUT is placed for operating as an energy harvester. Figure 5C,D show the RMS values of the voltage and power output, respectively. These measurements were carried out while connecting at the terminals of the pMUT a resistor load of 9 kΩ, a value corresponding to the pMUT impedance at the resonant frequency.

The following, Table 3, resumes the maximum RMS values obtained in the experiments as well as the calculation of sensitivity and the efficiency of the UPD system.

For the calculation of the effective values of power input, the values of voltage input were experimentally measured with the oscilloscope probe connected to the ends of the TX. Then, the square of the input voltage was divided by the equivalent input impedance, which was given by the parallel connection of the impedance of the oscilloscope and the impedance of the TX.

## 4. Discussion

Wireless energy transfer strategies are attracting more and more attention as alternative and reliable power sources, and the UPD systems can represent a cutting-edge source of energy to ensure extended operating time for IMDs [40,41].

Most of the studies in the literature indicate that the geometrical properties of TX and RX must be the same to optimize the amount of energy transfer in a UPD system [21]. However, in many applications, it is not possible because of constraints given by the shapes of human body and organs.

In the proposed work, the RX has smaller dimension than TX. The TX is a plate piezoelectric element, while the RX is a diaphragm-based structure. However, a plate piezoelectric structure comprises more piezo material than a diaphragm structure, thus resulting in a greater energy generation when it is excited from outside, and the diaphragm RX structure used allowed us to operate at a frequency lower than 70 kHz, thereby reducing the heating of tissue and having less attenuation of signal inside the body.

The choice of using the AlN piezoelectric material to develop the pMUT was made because it is non-toxic compared to the lead zirconate titanate (PZT) ceramic material, which is commonly used for the fabrication of most of the piezoelectric transducers [42]. Although AlN has a lower electromechanical coupling coefficient than PZT, it has the advantages of low-temperature deposition and low residual stress, which favor the complementary metal oxide semiconductor (CMOS) technology [43].

A simulation of the UPD system was carried out to verify the obtained experimental results. A one-dimensional (1D) lumped parameter transmission line model was implemented in the MATLAB^®^ environment. The six blocks representing the UPD system, which are shown in Figure 1, are depicted as a cascade of two-port networks using ABCD matrixes [44,45]. About the creation of the ABCD matrixes, see Supplementary Materials.

**Figure 6:**
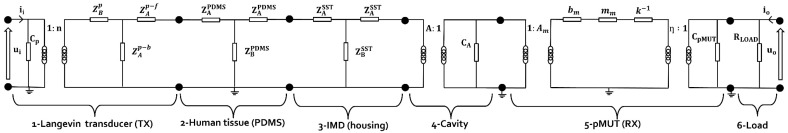
Entire lumped parameter transmission line model used to simulate the UPD system.

The resulting model is shown in Figure 6, and the relation between the input and output data is given by the following system of equations:

(3) $$\{\begin{array}{c} u_{i}=A\;u_{o}-B\;i_{o}\\ i_{i}=C\;u_{o}-D\;i_{o} \end{array}$$

The simulated power output is:

(4) $$P_{o}=\left|\frac{u_{o}^{2}}{R_{\mathrm{LOAD}}}\right|=\left|u_{i}^{2}R_{\mathrm{LOAD}}{\left({A\;R}_{\mathrm{LAOD}}-B\right)}^{-2}\right|$$

To obtain the RMS value for the simulated power output, the resulting $P_{o}$ value was then divided by factor $\sqrt{2}$.

The following, Table 4, compares the values of power output measured experimentally, with the simulated ones. In simulation, the excitation frequency, for which the power output reaches the maximum value, is approximately 66.963 kHz.

**Figure 7:**
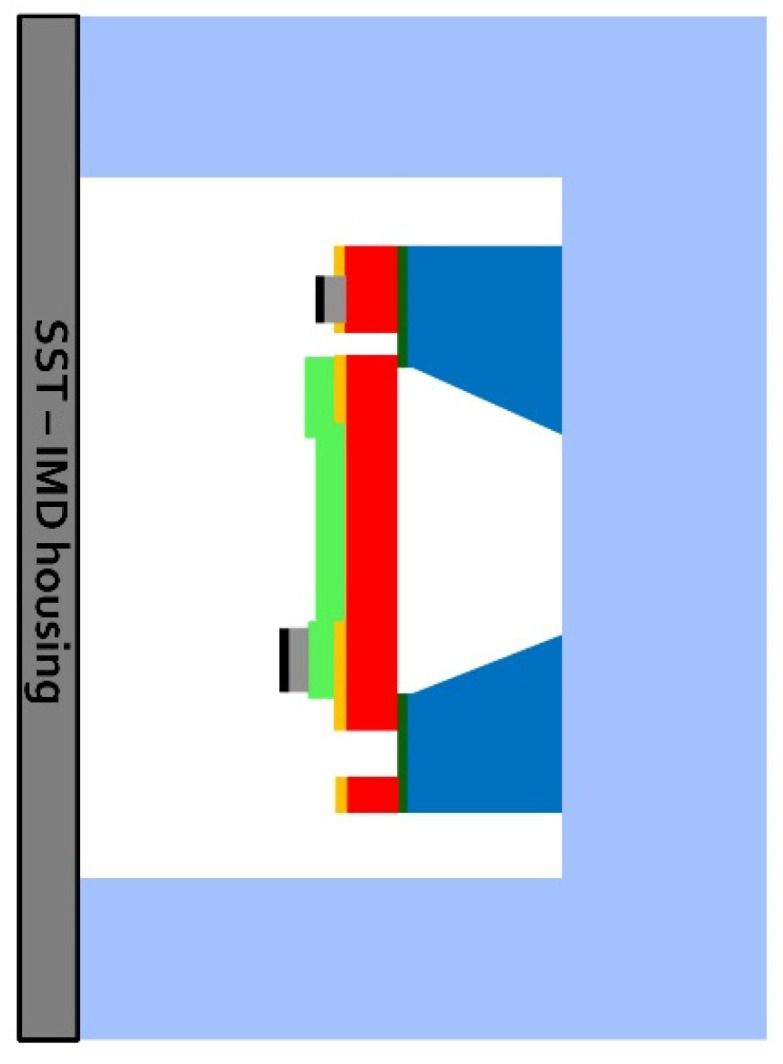
Schematic illustration of the pMUT within the cavity (dimensions are not in scale).

To highlight how the pMUT is installed in the receiver housing, Figure 7 shows an enlarged illustration of the pMUT placement.

In the modeling of the UPD system shown in Figure 6, the pMUT is placed in the coupling cavity. The effect of the cavity on the pMUT performance is considered by introducing the acoustic compliance, $C_{A}$. The following Table 5 resumes the values of the lumped parameters for the coupling cavity and the pMUT.

The results obtained while running the simulation program well reflect the measurements carried out experimentally.

The measured RMS values of power density, which are ranging from 0.1 to 21.6 µW cm^−2^ when the RMS input voltage varies from 1.768 to 35.355 V, are like those found in the literature [46]. By considering the short length of the pMUT side, i.e., 1.5 mm, many pMUT may be linked together to form a centimeter square array, thus greatly increasing the power output. In the literature, Sun et al. [47] proposed a piezo RX with flat-concave shape. The RX diameter is 20 mm, and it comprises an array of parallel-connected oscillators with diverse thicknesses. In this way, they obtained a wide band-range of frequency reception within 0.6 to 1.2 MHz. In such a frequency range, the RX answer was flat and, for an acoustic pressure of 1 kPa, they measured a power output of approximately 2.5 mW across a 160 Ω electrical load. However, they performed experiments by placing the sensors in a tank filled by degassed water, which is the case where losses are negligible. Shi et al. [24] proposed an array of PZT-diaphragms able to operate in a wide frequency band to avoid the effect of standing waves. By adjusting the excitation frequency of the input ultrasound, the power harvested by the UPD system can be easily increased for any given distance between the TX and RX. Their solution solves the issue of unpredictable power output for power transfer applications in the near field. He et al. [48] investigated the behavior of a UPD system in biological tissue when the excitation frequency is around 40 kHz. In such a study, researchers employ a piezoelectric thick film as RX with cross-section area of 30 mm^2^. At a given distance of 22 mm from the TX, the peak power measured was 49 μW for an input power of 51 mW.

Between solutions to develop lead-free pMUT, Joseph et al. [49] proposed a silk-based piezoelectric thin film operating at a resonant frequency of around 77 kHz with bandwidth of 2.44 kHz while it was characterized in air. Again, potassium-sodium niobate (KNN) is a lead-free piezoelectric material investigated to develop ultrasound wireless energy harvesting solutions for potential retinal electrical stimulation, which reached a peak-to-peak voltage output of 0.2 V when the peak-to-peak voltage input was set to 30 V [50]. Conceptual investigation at the interface between wireless power devices for a retina CMOS neuron integrated circuit was also carried out to support medical professionals in achieving an interfacing approach to restore the image visualization in people with neurodegenerative diseases [51]. Again, combination of pMUTs can be used to have abilities for the computation of mechanical logic operations in the design of systems for acoustic communication [52], and pMUTs can operate in chaotic regime for cryptographic applications in order to secure wireless data communications [53].

Regarding safety limits for the body exposed to low-frequency ultrasound (20–100 kHz [54]), Bocaud et al. [55] stated that the threshold to produce observable lesions in human skin were determined to be 2500 mW cm^−2^ at 20 kHz for an hour exposure to pulsed ultrasound and 10 min exposure to continuous wave in vitro.

The following, Table 6, summarizes advantages and drawbacks between ultrasound and electromagnetic solutions, while Table 7 lists similar results found in the literature regarding solutions using ultrasound.

Regarding a practical application for charging an IMD, such as a cardiac pacemaker that requires an energy of about 15 µJ to operate [66], the proposed UPD system can harvest such an amount of energy in 2 h for the minimum value of measured power output (2 nW), while only 31 s for the maximum measured power output value (486 nW).

To conclude, wireless energy transfer is convenient for charging the small batteries of IMDs. As is visible in Table 6, electromagnetic radiation is optimal for relatively large IMDs, e.g., the cm- and sub-cm scales, at short distance. Conversely, for charging mm sized IMDs in depth, ultrasound is advantageous because of low losses in tissues, and low acoustic velocities allow operation at lower frequencies.

Among the results shown in Table 7, AlN-based pMUTs are less efficient than PZT-based ones, but they are lead-free and CMOS-compatible.

The proposed solution describes a UPD system operating at 67 kHz, i.e., low-frequency ultrasound, while other AlN-based pMUTs operate in the range between hundreds of kHz and MHz. Moreover, our solution tries to mimic a real situation where the pMUT is integrated in a metallic case and the propagating medium is PDMS material. The PDMS material has the most skin-like properties in comparison with the propagation media used by other authors, e.g., water, oil, and air. In future studies, it will be useful to plan using animal tissues to be as close to real human tissue characteristics as possible.

Again, the pMUT RX used in the proposed study is fabricated with a mature technology process that can be readily applied for further development. Consequently, the receiver data used for the system evaluation are realistic and increase the estimation accuracy.

## 5. Conclusions

In this work, a UPD system was modeled and experimentally tested to measure the amount of power transferred for implantable device charging applications without undergoing surgery. The UPD system comprises a Langevin TX, a PDMS layer to mimic the human tissues, a thin layer of Type 316L SST as IMD housing, a square AlN-based pMUT used as RX, and a resistive load to optimize the amount of transferred power. At an excitation frequency of approximately 66.9 kHz, for an RMS voltage input of approximately 35 V, the resulting power density was 21.6 µW cm^−2^. The data measured experimentally were congruent with the data obtained by simulation.

## Figures and Tables

**Table 1:** Values of parameters characterizing the piezoelectric material in both TX and RX.

Parameter	PZT-8 (TX)	AlN (RX)
Cross-section Area (S) [m^2^]	9.1185·10^−4^	2.25·10^−6^
Thickness (t) [m]	5.75·10^−3^	5·10^−7^
Length (l) [m]	-	1.5·10^−3^
Density (ρ) [kg m^−3^]	7600	3260
Young’s Modulus (E) [Pa]	7.4·10^10^	3.45·10^11^
Poisson’s Ratio (υ)	-	0.32
Plate Modulus (Y) [Pa]	-	3.8436·10^11^
Dielectric Constant (ε)	1200	7.9059
Piezo Charge Coeff. (d_31_, d_33_) [pC N^−1^]	-, 240	−2.3259, -
Piezo Constant (e_31_, e_33_) [C m^−2^]	-, 14.6385	−0.8024, -
Coupling Factor (k)	0.48	0.17
Quality Factor (Q)	1000	231 *^1^*
Dielectric Loss (tan δ′)	0.004	0.001

*^1^* Experimentally calculated with the pMUT in transmitter mode (see Supplementary Materials).

**Table 2:** Parameter values of all layers constituting the pMUT (RX).

Parameter	Si Layer	Dioxide (SiO_2_)	AlN Diaphragm	Al Pad	Cr Pad
Cross-section Area (S) [m^2^]	2.25·10^−6^	2.25·10^−6^	2.25·10^−6^	2.25·10^−6^	2.25·10^−6^
Thickness (t) [m]	10·10^−6^	2·10^−7^	5·10^−7^	1·10^−6^	20·10^−9^
Length (l) [m]	1.5·10^−3^	1.5·10^−3^	1.5·10^−3^	1.5·10^−3^	1.5·10^−3^
Volume (V) [m^3^]	2.25·10^−11^	4.5·10^−13^	1.125·10^−12^	2.25·10^−12^	4.5·10^−14^
Density (ρ) [kg m^−3^]	2330	2200	3260	2680	7140
Mass (M) [kg]	5.243·10^−8^	9.9·10^−10^	3.668·10^−9^	6.03·10^−9^	3.213·10^−10^
Young’s Modulus (E) [Pa]	1.65·10^11^	0.73·10^11^	3.45·10^11^	0.71·10^11^	2.45·10^11^
Poisson’s Ratio (υ)	0.22	0.17	0.32	0.33	0.2
Plate Modulus (Y) [Pa]	1.734·10^11^	0.752·10^11^	3.844·10^11^	0.797·10^11^	2.552·10^11^
Mid-plane Location (z) [m]	5·10^−6^	1.01·10^−5^	1.045·10^−5^	1.12·10^−5^	1.171·10^−5^
Top-plane Location (h) [m]	1·10^−5^	1.02·10^−5^	1.07·10^−5^	1.17·10^−5^	1.172·10^−5^

**Table 3:** Experimentally obtained results for the UPD system.

RMS Input Voltage (V)	RMS Acoustic Pressure (Pa)	RMS Voltage Output (mV)	Sensitivity V_OUT_/V_IN_ (%)	Power Input (mW cm^−2^)	Power Output (µW cm^−2^)	Efficiency P_OUT_/P_IN_ (%)
1.768	6.5	2.5	0.141	0.97	0.1	0.010
7.071	25.5	9.6	0.136	15.72	1.4	0.009
17.678	59.4	21.2	0.120	91.70	6.7	0.007
35.355	105.6	38.1	0.108	372.50	21.6	0.006

**Table 4:** Comparison between measured and simulated RMS power output values.

RMS Input Voltage (V)	Measured RMS Power Output (nW)	Simulated RMS Power Output (nW)
1.768	2	3
7.071	31	35
17.678	150	158
35.355	486	497

**Table 5:** Values of the lumped parameters for the coupling cavity and the pMUT.

Parameter	Value
SST housing cross-sectional area (A) [m^2^]	1·10^−4^
Acoustic compliance (C_A_) [m^5^ N^−1^]	7.85·10^−13^
Back-air cavity cross-sectional area (*A_m_*) [m^2^]	0.75·10^−6^
Damping (*b_m_*) [N s m^−1^]	9.09·10^−6^
Mass (*m_m_*) [kg]	5.01·10^−9^
Compliance (*k*^−1^) [m N^−1^]	1.16·10^−3^
Coupling coefficient (η) [N V^−1^]	8.31·10^−5^
Equivalent capacitance (C_pMUT_) [F]	290·10^−12^

**Table 6:** Advantages and drawbacks between ultrasound and electromagnetic solutions.

Solution	Advantages	Disadvantages	Ref.
Electromagnetic (far field)	Small antenna	Low efficiency	[56]
		High loss in body	[57]
Electromagnetic (near field)	High performance	Short distance	[58]
	Low loss in body	Coil size	[59]
Ultrasound	High efficiency in body	High loss in air	[60]
	Deeply implant	Special equipment	[61]

**Table 7:** Comparison between ultrasound pMUT solutions.

RX Struct.	RX csa (mm^2^)	TX-RX Distan. (mm)	Propagat. Medium	Oper. Freq. (kHz)	Power Input (mW cm^−2^)	Power Output (µW cm^−2^)	Effic. (%)	Lead Free	CMOS Comp.	Ref.
PZT	2.25	23	water	350	65	4.1	0.0063	NO	NO	[62]
PZT	2.058	10	water	240	1	3.75	0.375	NO	NO	[24]
PZT	4	20	water	88	322	1063	0.33	NO	NO	[35]
PZT	314	-	water	wide range 600–1200	-	-	-	NO	NO	[47]
PNZT	30	22	fatty	40	-	-	0.096	NO	NO	[48]
KNNS	2	20	water	304	-	-	-	YES	NO	[50]
AlN	16	40	oil	2000	77	71	0.009	YES	YES	[63]
AlN	1.44	127	air	500	-	-	-	YES	YES	[64]
AlN	2.55	25	water	3000	7	16.47	0.235	YES	YES	[65]
AlN	2.25	8.6	PDMS	67	1	0.1	0.010	YES	YES	This work

RX: receiver; TX: transmitter; csa: cross-sectional area; PZT: lead zirconate titanate; PNZT: niobium-doped lead zirconate titanate; KNNS: (K_0.48_Na_0.52_)(Nb_0.95_Sb_0.05_)O_3_(Bi_0.4_La_0.1_)(Na_0.4_Li_0.1_)ZrO_3_; AlN: aluminum nitride; PDMS: polydimethylsiloxane.

## Data Availability

The data that support the findings of this study are available from A.P. (Antonino Proto) upon reasonable request.

## Supplementary Materials

The following supporting information can be downloaded at: https://www.mdpi.com/article/10.3390/mi13122127/s1. References [67,68,69,70,71,72,73,74] are cited in the Supplementary Materials file.

## References

[1] Amar A.B., Kouki A.B., Cao H. (2015). Power approaches for implantable medical devices. Sensors.

[2] Sheng H., Zhang X., Liang J., Shao M., Xie E., Yu C., Lan W. (2021). Recent Advances of Energy Solutions for Implantable Bioelectronics. Adv. Healthc. Mater..

[3] Sette A.L., Seigneuret E., Reymond F., Chabardes S., Castrioto A., Boussat B., Moro E., Francois P., Fraix V. (2019). Battery longevity of neurostimulators in Parkinson disease: A historic cohort study. Brain Stimul..

[4] Alam M.B., Munir M.B., Rattan R., Flanigan S., Adelstein E., Jain S., Saba S. (2014). Battery longevity in cardiac resynchronization therapy implantable cardioverter defibrillators. Europace.

[5] Ortiz-Catalan M. (2020). Ultrasound-powered tiny neural stimulators. Nat. Biomed. Eng..

[6] Chakrabartty S., Lajnef N., Elvin N.G., Elvin A., Iniewski K. (2008). Toward Self-Powered Sensors and Circuits for Biomechanical Implants. VLSI Circuits for Biomedical Applications.

[7] Zebda A., Alcaraz J.-P., Vadgama P., Shleev S., Minteer S.D., Boucher F., Cinquin P., Martin D.K. (2018). Challenges for successful implantation of biofuel cells. Bioelectrochemistry.

[8] Thimot J., Shepard K.L. (2017). Bioelectronic devices: Wirelessly powered implants. Nat. Biomed. Eng..

[9] Marketing Clearance of Diagnostic Ultrasound Systems and Transducers. https://www.fda.gov/media/71100/download.

[10] Guidelines for Evaluating the Environmental Effects of Radio Frequency Radiation. https://transition.fcc.gov/Bureaus/Engineering_Technology/Orders/1996/fcc96326.pdf.

[11] IEEE Standard for Safety Levels with Respect to Human Exposure to Radio Frequency Electromagnetic Fields, 3 kHz to 300 GHz. https://ieeexplore.ieee.org/stamp/stamp.jsp?arnumber=1626482.

[12] Harvey G., Gachagan A., Mutasa T. (2014). Review of high-power ultrasound-industrial applications and measurement methods. IEEE Trans. Ultrason. Ferroelectr. Freq. Control.

[13] Medan M.S., Abd El-Aty A.M. (2010). Advances in ultrasonography and its applications in domestic ruminants and other farm animals reproduction. J. Adv. Res..

[14] Eshky A., Cleland J., Ribeiro M.S., Sugden E., Richmond K., Renals S. (2021). Automatic audiovisual synchronisation for ultrasound tongue imaging. Speech Commun..

[15] Ozeri S., Shmilovitz D. (2010). Ultrasonic transcutaneous energy transfer for powering implanted devices. Ultrasonics.

[16] Mazzilli F., Lafon C., Dehollain C. (2014). A 10.5 cm ultrasound link for deep implanted medical devices. IEEE Trans. Biomed. Circuits Syst..

[17] Radziemski L., Makin I.R.S. (2016). In vivo demonstration of ultrasound power delivery to charge implanted medical devices via acute and survival porcine studies. Ultrasonics.

[18] Huang Y., Zhao J., Sun W., Yang H., Liu Y. (2019). Investigation and Modeling of Multi-Node Body Channel Wireless Power Transfer. Sensors.

[19] Chang T.C., Weber M.J., Charthad J., Baltsavias S., Arbabian A. (2018). End-to-end design of efficient ultrasonic power links for scaling towards submillimeter implantable receivers. IEEE Trans. Biomed. Circuits Syst..

[20] Arbabian A., Chang T.C., Wang M.L., Charthad J., Baltsavias S., Fallahpour M., Weber M.J. (2016). Sound Technologies, Sound Bodies. IEEE Microw. Mag..

[21] Basaeri H., Christensen D.B., Roundy S. (2016). A review of acoustic power transfer for bio-medical implants. Smart Mater. Struct..

[22] Laursen K., Rashidi A., Hosseini S., Mondal T., Corbett B., Moradi F. (2020). Ultrasonically Powered Compact Implantable Dust for Optogenetics. IEEE Trans. Biomed. Circuits Syst..

[23] Chang T.C., Weber M.J., Wang M.L., Charthad J., Khuri-Yakub B.T., Arbabian A. (2016). Design of Tunable Ultrasonic Receivers for Efficient Powering of Implantable Medical Devices With Reconfigurable Power Loads. IEEE Trans. Ultrason. Ferroelectr. Freq. Control.

[24] Shi Q., Wang T., Lee C. (2016). MEMS Based Broadband Piezoelectric Ultrasonic Energy Harvester (PUEH) for Enabling Self-Powered Implantable Biomedical Devices. Sci. Rep..

[25] Eovino B.E., Liang Y., Lin L. Concentric PMUT Arrays for Focused Ultrasound and High Intensity Applications. Proceedings of the IEEE International Conference on Micro Electro Mechanical Systems (MEMS).

[26] Wang L., Chiu Y., Gong D., Ma S., Yang Y., Li H., Lee H., Liu H., Jin Y. Fabrication Process and Performance Analysis of AlN based Piezoelectric Micromachined Ultrasonic Transducer with a Suspended Structure. Proceedings of the 14th Annual IEEE International Conference on Nano/Micro Engineered and Molecular Systems (NEMS).

[27] Alasatri S., Rufer L., Lee J.E.-Y. (2018). AlN-on-Si Square Diaphragm Piezoelectric Micromachined Ultrasonic Transducer with Extended Range of Detection. Proceedings.

[28] Sammoura F., Shelton S., Akhbari S., Horsley D., Lin L. A Two-Port Piezoelectric Micromachined Ultrasonic Transducer. Proceedings of the Joint IEEE International Symposium on the Applications of Ferroelectric, International Workshop on Acoustic Transduction Materials and Devices and Workshop on Piezoresponse Force Microscopy (ISAF/IWATMD/PFM).

[29] Zhu Q., Chen T., Liu H., Sun L., Wang T., Lee C., Le X., Xie J. An AlN-based piezoelectric micro-machined ultrasonic transducer (pMUT) array. Proceedings of the 16th IEEE International Conference on Nanotechnology (IEEE NANO).

[30] Halbach A., Gijsenbergh P., Jeong Y., Billen M., Chare C., Gao H., Torri G.B., Cheyns D., Rottenberg X., Rochus V. Modelling of display-compatible piezoelectric micromachined ultrasonic transducers for haptic feedback. Proceedings of the 20th International Conference on Thermal, Mechanical and Multi-Physics Simulation and Experiments in Microelectronics and Microsystems (EuroSimE).

[31] Huang C.H., Demi L., Torri G.B., Mao S., Billen M., Jeong Y., Cheyns D., Rottenberg X., Rochus V. Display compatible pMUT device for mid air ultrasound gesture recognition. Proceedings of the 11th Annual TechConnect World Innovation Conference and Expo, Held Jointly with the 20th Annual Nanotech Conference and Expo, the SBIR/STTR Spring Innovation Conference, and the Defense TechConnect DTC Spring Conference.

[32] Griffin B.A., Williams M.D., Coffman C.S., Sheplak M. (2011). Aluminum nitride ultrasonic air-coupled actuator. J. Microelectromechanical Syst..

[33] Przybyla R.J., Shelton S.E., Guedes A., Izyumin I.I., Kline M.H., Horsley D.A., Boser B.E. (2011). In-air rangefinding with an AlN piezoelectric micromachined ultrasound transducer. IEEE Sens. J..

[34] Alasatri S., Mak K.L., Benserhir J., Lee J.E.-Y., Rufer L. Air-coupled Ultrasonic Rangefinder with Meter-long Detection Range Based on a Dual-electrode PMUT Fabricated Using a Multi-user MEMS Process. Proceedings of the 18th IEEE Sensors Conference.

[35] Basaeri H., Yu Y., Young D., Roundy S. (2019). A MEMS-Scale Ultrasonic Power Receiver for Biomedical Implants. IEEE Sens. Lett..

[36] Rebelo R., Barbosa A.I., Correlo V.M., Reis R.L. (2021). An outlook on implantable biosensors for personalized medicine. Engineering.

[37] Horsley D.A., Rozen O., Lu Y., Shelton S., Guedes A., Przybyla R., Tang H.-Y., Boser B.E. Piezoelectric Micromachined Ultrasonic Transducers for Human-Machine Interfaces and Biometric Sensing. Proceedings of the IEEE Sensors Conference.

[38] Cowen A., Hames G., Glukh K., Hardy B. (2013). PiezoMUMPs Design Handbook.

[39] Bolt Clamped Langevin Transducer 40 KHz. https://www.steminc.com/PZT/en/bolt-clamped-langevin-tranducer-40-khz.

[40] Yoo S., Lee J., Joo H., Sunwoo S.-H., Kim S., Kim D.-H. (2021). Wireless Power Transfer and Telemetry for Implantable Bioelectronics. Adv. Healthc. Mater..

[41] Khan S.R., Pavuluri S.K., Cummins G., Desmulliez M.P.Y. (2020). Wireless power transfer techniques for implantable medical devices: A review. Sensors.

[42] Todar M.T., Guido F., Algieri L., Mastronardi V.M., Desmaele D., Epifani G., De Vittorio M. (2018). Biocompatible, flexible, and compliant energy harvesters based on piezoelectric thin films. IEEE Trans. Nanotechnol..

[43] Fei C., Liu X., Zhu B., Li D., Yang X., Yang Y., Zhou Q. (2018). AlN piezoelectric thin films for energy harvesting and acoustic devices. Nano Energy.

[44] Peres P.L.D., de Souza C.R., Bonatti I.S. (2003). ABCD matrix: A unique tool for linear two-wire transmission line modelling. Int. J. Electr. Eng. Educ..

[45] Frickey D.A. (1994). Conversions between S, Z, Y, H, ABCD, and T parameters which are valid for complex source and load impedances. IEEE Trans. Microw. Theory Tech..

[46] Turner B.L., Senevirathne S., Kilgour K., McArt D., Biggs M., Menegatti S., Daniele M.A. (2021). Ultrasound-Powered Implants: A Critical Review of Piezoelectric Material Selection and Applications. Adv. Healthc. Mater..

[47] Sun Y., Gao X., Wang H., Chen Z., Yang Z. (2018). A wideband ultrasonic energy harvester using 1-3 piezoelectric composites with non-uniform thickness. Appl. Phys. Lett..

[48] He Q., Liu J., Yang B., Wang X., Chen X., Yang C. (2014). MEMS-based ultrasonic transducer as the receiver for wireless power supply of the implantable microdevices. Sens. Actuator A Phys..

[49] Joseph J., Singh S.G., Vanjari S.R.K. (2018). Piezoelectric Micromachined Ultrasonic Transducer Using Silk Piezoelectric Thin Film. IEEE Electron. Device Lett..

[50] Jiang L., Yang Y., Chen R., Lu G., Li R., Xing J., Shung K.K., Humayun M.S., Zhu J., Chen Y. (2019). Ultrasound-induced wireless energy harvesting for potential retinal electrical stimulation application. Adv. Funct. Mater..

[51] Al-Shidaifat A., Kumar S., Chakrabartty S., Song H.J. (2020). A Conceptual Investigation at the Interface between Wireless Power Devices and CMOS Neuron IC for Retinal Image Acquisition. Appl. Sci..

[52] Liu X., Chen D., Yang D., Chen X., Le X., Xie J. (2019). A Computational Piezoelectric Micro-Machined Ultrasonic Transducer Toward Acoustic Communication. IEEE Electron. Device Lett..

[53] Defoort M., Rufer L., Fesquet L., Basrour S. (2021). A dynamical approach to generate chaos in a micromechanical resonator. Microsyst. Nanoeng..

[54] Ahmadi F., McLoughlin I.V., Chauhan S., ter-Haar G. (2012). Bio-effects and safety of low-intensity, low-frequency ultrasonic exposure. Prog. Biophys. Mol. Biol..

[55] Boucaud A., Montharu J., Machet L., Arbeille B., Machet M.C., Patat F., Vaillant L. (2001). Clinical, histologic, and electron microscopy study of skin exposed to low-frequency ultrasound. Anat. Rec..

[56] Kim S., Ho J.S., Chen L.Y., Poon A.S.Y. (2012). Wireless power transfer to a cardiac implant. Appl. Phys. Lett..

[57] Kazanc O., Maloberti F., Dehollain C. Simulation oriented rectenna design methodology for remote powering of wireless sensor systems. Proceedings of the IEEE International Symposium on Circuits and Systems (ISCAS).

[58] Ko Y.Y., Ho S.L., Fu W.N., Zhang X. (2012). A novel hybrid resonator for wireless power delivery in bio-implantable devices. IEEE Trans. Magn..

[59] Cong P., Suster M.A., Chaimanonart N., Young D.J. Wireless power recharging for implantable bladder pressure sensor. Proceedings of the IEEE Sensors Conference (SENSORS).

[60] Sanni A., Vilches A., Toumazou C. (2012). Inductive and ultrasonic multi-tier interface for low-power, deeply implantable medical devices. IEEE Trans. Biomed. Circuits Syst..

[61] Mazzilli F., Thoppay P.E., Praplan V., Dehollaini C. Ultrasound energy harvesting system for deep implanted-medical-devices (IMDs). Proceedings of the IEEE International Symposium on Circuits and Systems (ISCAS).

[62] Jiang L., Yang Y., Chen R., Lu G., Li R., Li D., Humayun M.S., Shung K.K., Zhu J., Chen Y. (2019). Flexible piezoelectric ultrasonic energy harvester array for bio-implantable wireless generator. Nano Energy.

[63] Herrera B., Pop F., Cassella C., Rinaldi M. AlN PMUT-based Ultrasonic Power Transfer Links for Implantable Electronics. Proceedings of the 20th International Conference on Solid-State Sensors, Actuators and Microsystems and Eurosensors XXXIII, TRANSDUCERS 2019 and EUROSENSORS XXXIII.

[64] Gong D., Ma S., Chiu Y., Lee H., Jin Y. Study of the properties of AlN PMUT used as a wireless power receiver. Proceedings of the IEEE 69th Electronic Components and Technology Conference (ECTC).

[65] Rong Z., Zhang M., Ning Y., Pang W. (2022). An ultrasound-induced wireless power supply based on AlN piezoelectric micromachined ultrasonic transducers. Sci. Rep..

[66] Moerke C., Wolff A., Ince H., Ortak J., Oner A. (2022). New strategies for energy supply of cardiac implantable devices. Herzschr. Elektrophys..

[67] Zhang J.-G., Long Z.-L., Ma W.-J., Hu G.-H., Li Y.-M. (2019). Electromechanical dynamics model of ultrasonic transducer in ultrasonic machining based on equivalent circuit approach. Sensors.

[68] Sherrit S., Leary S.P., Dolgin B.P., Bar-Cohen Y. Comparison of the Mason and KLM equivalent circuits for piezoelectric resonators in the thickness mode. Proceedings of the IEEE Ultrasonics Symposium.

[69] Kim J., Lee J. (2019). Theoretical Resonance Analysis of Langevin Transducers with Equivalent Circuit Models for Therapeutic Ultrasound. J. Electr. Eng. Technol..

[70] Meeker T.R. (1996). Publication and proposed revision of ANSI/IEEE standard 176-1987 “ANSI/IEEE standard on piezoelectricity”. IEEE Trans. Ultrason. Ferroelectr. Freq. Control.

[71] Uchino K. (2017). High-Power Piezoelectrics and Loss Mechanisms. Advanced Piezoelectric Materials: Science and Technology.

[72] Ashby M.F. (2011). Material Property Charts. Materials Selection in Mechanical Design.

[73] Horsley D., Lu Y., Rozen O., Bhurgra H., Piazza G. (2017). Flexural Piezoelectric Resonators. Piezoelectric MEMS Resonators.

[74] Marzencki M., Basrour S., Choudhary V., Iniewski K. (2013). Modeling of Piezoelectric MEMS Vibration Energy Harvesters. MEMS: Fundamental Technology and Applications.

