# Supplementary material for: Modeling and Measurement of an Ultrasound Power Delivery System for Charging Implantable Devices Using an AlN-Based pMUT as Receiver
Source: Micromachines (Basel). 2022 Dec 1;13(12):2127. doi: 10.3390/mi13122127 (PMC9788435; doi:10.3390/mi13122127)
Supplement: Supplementary file 1 [file micromachines-13-02127-s001.zip › micromachines-1967018-supplementary.docx]

Modelling and Measurement of an Ultrasound Power Delivery System for Charging Implantable Devices using an AlN-based pMUT as receiver

Antonino Proto, Libor Rufer, Skandar Basrour and Marek Penhaker

The quality factor value of the RX, $Q_{\mathrm{pMUT}}$, was obtained by following the ring-down methodology.

It is a rapid way to calculate the quality factor of resonators. Particularly, the resonator, excited from the waveform generator in the burst mode, will take a certain period to get his maximum value, as well as it will take the same period to go back to its off-state when the input burst ends. The formula for the calculation of $Q_{\mathrm{pMUT}}$ is:

|  | $Q_{\mathrm{pMUT}}=\pi f_{R} \tau$ | (1S) |
| --- | --- | --- |

where $\tau$ is the rate of decay for which the signal attenuates by a factor $1/e$ ≌ 37% (Figure S1). The resulting $Q_{\mathrm{pMUT}}$ value is approximately 231.

| 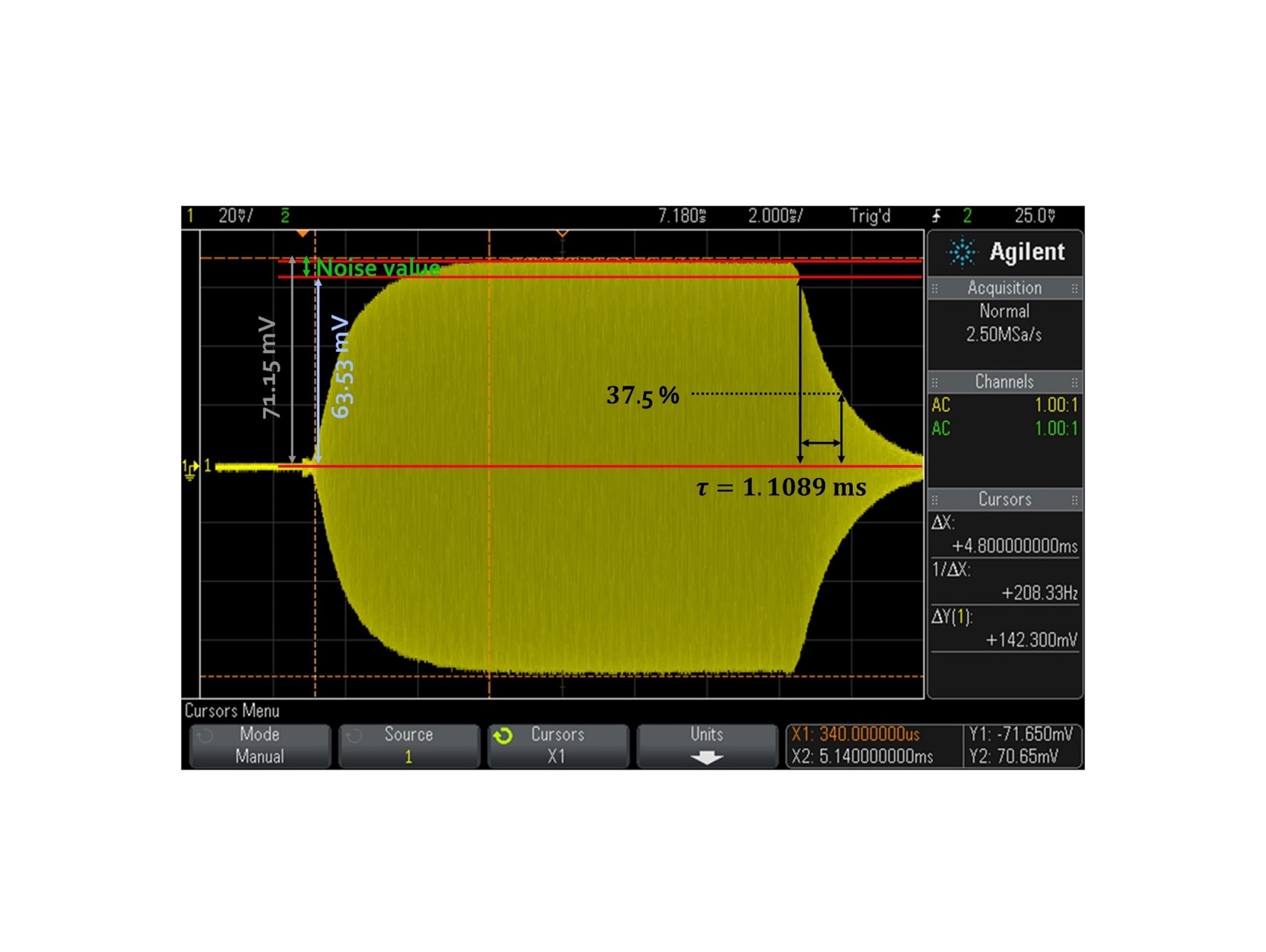 |
| --- |
| **Figure S1.** The pMUT excitation when the input is given by burst mode. |

In the following, the procedure to get the one-dimensional (1D) lumped parameter transmission line model for the UPD system.

The TX is the Langevin transducer. It works in the thickness mode. Thus, we can use as the first approximation a 1D-model using two-ports networks to describe the back side, the stack of piezoelectric plates, and the front side (Figure S2).

As it is visible in Figures S2(A-B), the back and piezo stack parts of the TX comprise also the screw bolt. These two parts have a uniform cross-section, and each impedance is computed by using the following equations [1]:

|  | $Z_{A}=j c \rho S_{0}\mathrm{tg}\left( \frac{k_{e} m t}{2} \right) ; Z_{B}=c \rho S_{0}\frac{1}{j sin\left( k_{e} m t \right)}$ | (2S); (3S) |
| --- | --- | --- |

In equations (2S) and (3S), $m$ is the number of stack elements (for the piezoelectric material there are two stacked plates connected in parallel), $\rho$ and $c$ are the density and speed of sound of materials, $k_{e}$ is the wave number, $t$ is the thickness and $S_{0}=\pi\left( r_{\mathrm{outer}}^{2}-r_{\mathrm{inner}}^{2} \right)$ is the cross-section area.

| 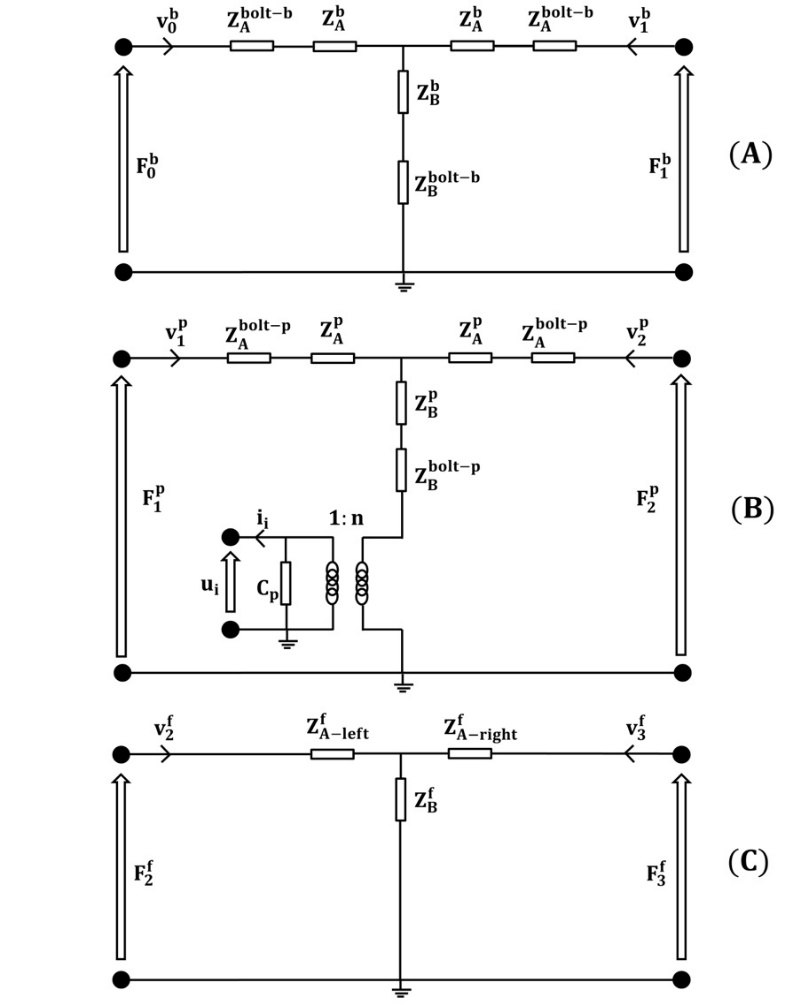 |
| --- |
| **Figure S2.** Two-ports network to describe the blocks of the TX. Back side (A); piezoelectric stack (B); front side (C). |

Figure S2(B) shows the Mason’s circuit used to model the piezoelectric element [2]. It consists of two mechanical ports and an electrical one related with the electromechanical transformer having the turns ratio value of $n$. On the mechanical side of the transformer is the capacitance $C_{p}$ of the piezoelectric plate. The values of $C_{p}$ and $n$ can be calculated from the following expressions [1, 3]:

|  | $C_{p}=m \varepsilon_{33}^{T}\frac{S_{0}}{t} ; n=\frac{d_{33}}{s_{33}^{E}}\frac{S_{0}}{t}$ | (4S); (5S) |
| --- | --- | --- |

where $d_{33}$ is the piezoelectric charge coefficient, $\varepsilon_{33}^{T}$ is the permittivity of the piezoelectric plate at a constant mechanical stress, and $s_{33}^{E}$ is the mechanical compliance of the plate at a constant electric field.

Figure S2(C) is the two-port network of the waveguide located on the front side of the piezoelectric stack. It has a non-uniform cross-section area, whose value varies along the direction of propagation of the wave, $x$, by following the expression:

|  | $S(x)=S_{0}e^{-2\gamma x}$ | (6S) |
| --- | --- | --- |

where in this case $S_{0}$ is the cross-section area at the end of the front side, and $\gamma$ is the decay coefficient:

|  | $\gamma=\frac{\ln(\alpha)}{t}$ | (7S) |
| --- | --- | --- |

The $\alpha$ value is the ratio between large and small radius, and $t$ in equation (7S) is the thickness of front side material. Thus, each impedance of Figure S2(C) is computed through the following equations [3]:

|  | $Z_{A-left}^{f}=j c \rho S_{0} \frac{k_{e}^{'}}{{\alpha^{2} k}_{e}} \left( \frac{\alpha-\cos(k_{e}^{'} t)}{\sin(k_{e}^{'} t)} \right)-\frac{\gamma}{k_{e}^{'}}$ | (8S) |
| --- | --- | --- |
|  | $Z_{A-right}^{f}=j c \rho S_{0} \frac{k_{e}^{'}}{k_{e}} \left( \frac{1-\alpha\cos(k_{e}^{'} t)}{\alpha\sin(k_{e}^{'} t)} \right)+\frac{\gamma}{k_{e}^{'}}$ | (9S) |
|  | $Z_{B}^{f}=c \rho S_{0}\frac{k_{e}^{'}}{j \alpha k_{e}\sin\left( k_{e}^{'} t \right)}$ | (10S) |
|  | $k_{e}^{'}=\sqrt{k_{e}^{2}-\gamma^{2}}$ | (11S) |

Figure S3 shows the resulting TX equivalent circuit formed by lumped elements.

| 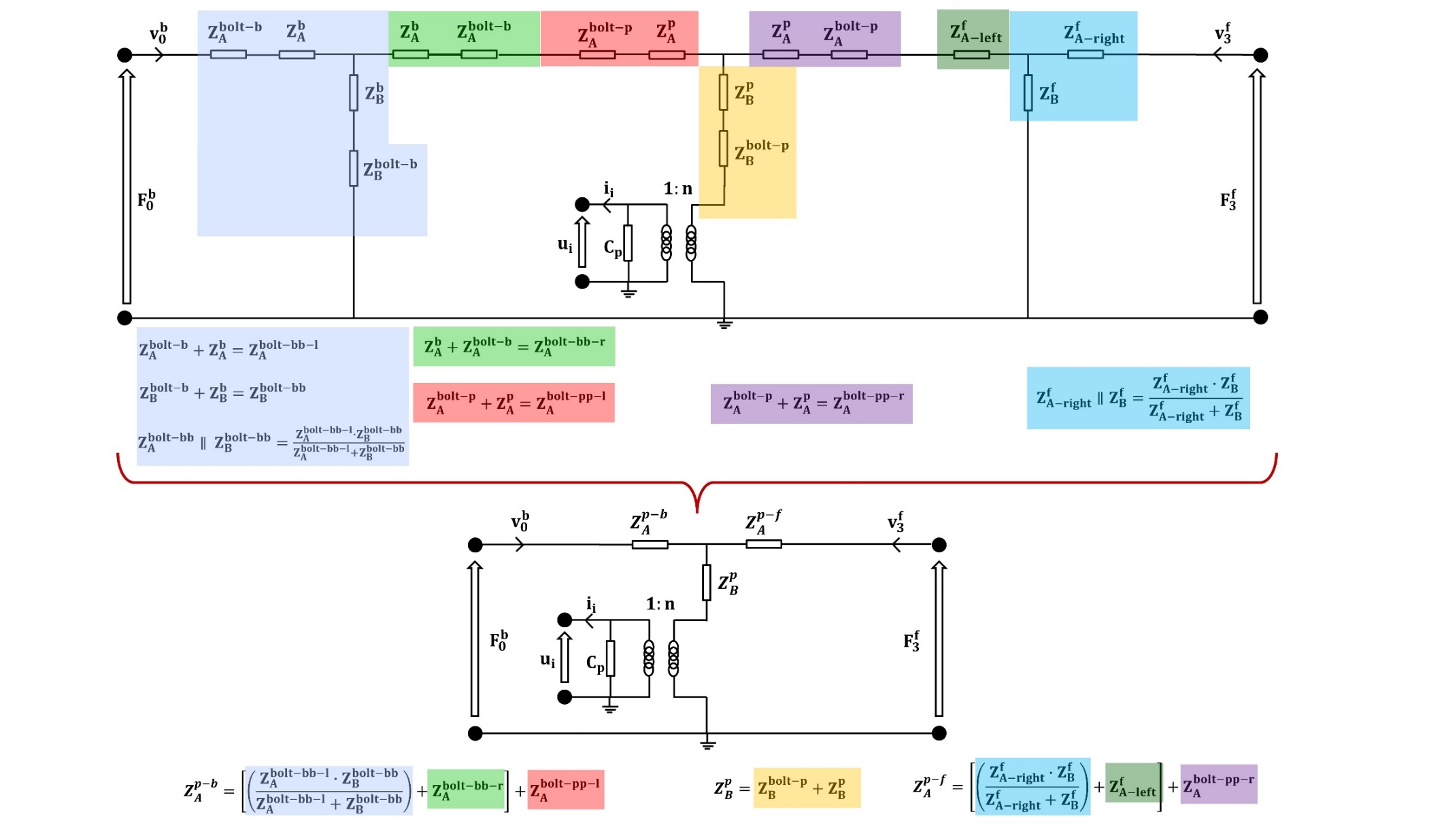 |
| --- |
| **Figure S3.** 1-D lumped element model of the complete TX, with its simplified resulting 3-ports Mason’s circuit. |

The resulting circuit in Figure S3, is the 3-ports model of the Mason’s circuit for a piezoelectric transducer working in the “33” mode. Assuming the back side of TX loaded with a low impedance of the air, the simplified 2-port model of the Mason’s circuit shown in Figure S4 will be used in the following analysis.

| 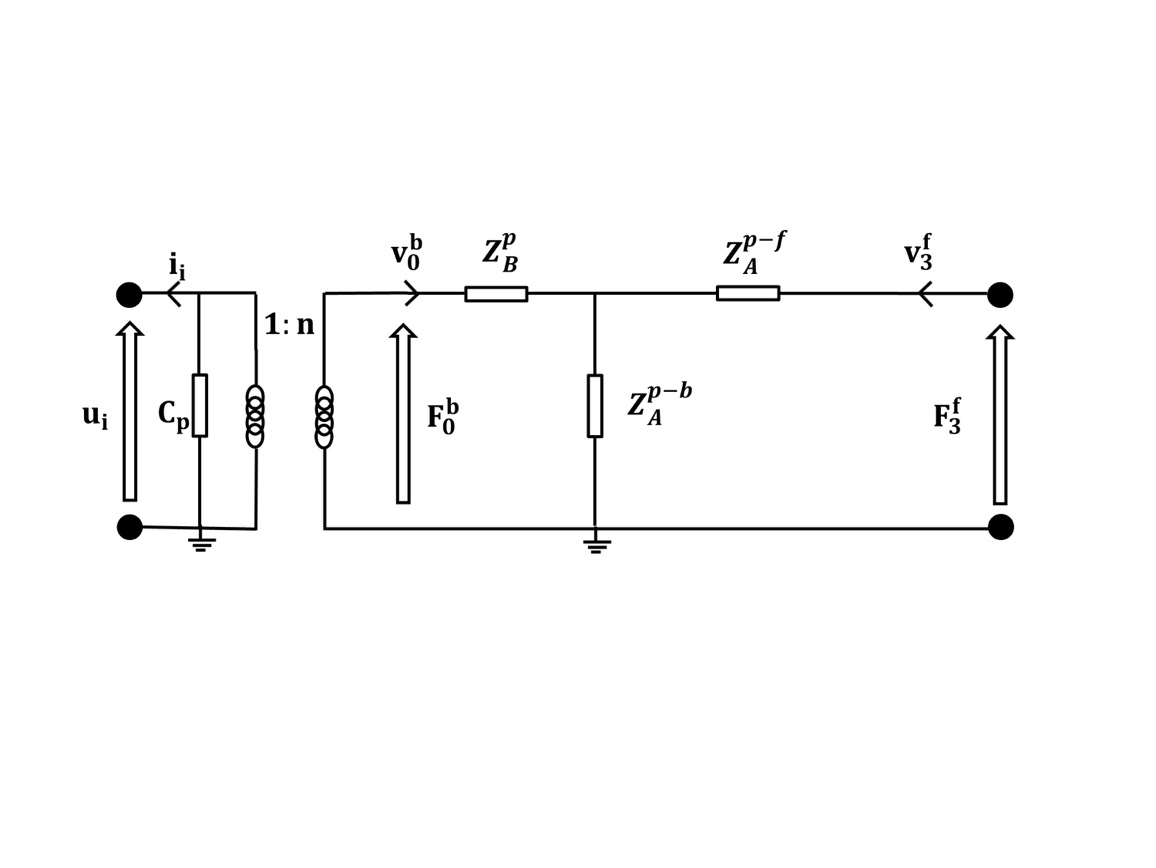 |
| --- |
| **Figure S4.** Equivalent 2-port model of the Mason’s circuit of the TX. |

To solve the circuit in Figure S4, the methodology based on the creation of ABCD matrixes is adopted in this study. Resulting equation for this 2-port network model is:

|  | $\left[ \begin{matrix} u_{i} \\ i_{i} \end{matrix} \right]=\left[ \mathrm{ABCD} \right]\left[ \begin{matrix} F_{3}^{f} \\ -v_{3}^{f} \end{matrix} \right]\Leftrightarrow\left[ \begin{matrix} u_{i} \\ i_{i} \end{matrix} \right]=\left[ \begin{matrix} A & B \\ C & D \end{matrix} \right]\left[ \begin{matrix} F_{3}^{f} \\ -v_{3}^{f} \end{matrix} \right]$ | (12S) |
| --- | --- | --- |
|  | $\Rightarrow\left\{ \begin{aligned} u_{i}=A F_{3}^{f}-B v_{3}^{f} \\ i_{i}=C F_{3}^{f}-D v_{3}^{f} \end{aligned} \right.$ |  |

To describe parameters of Figure S4, the ABCD matrixes must be created by following instructions given in Figure 5S.

| **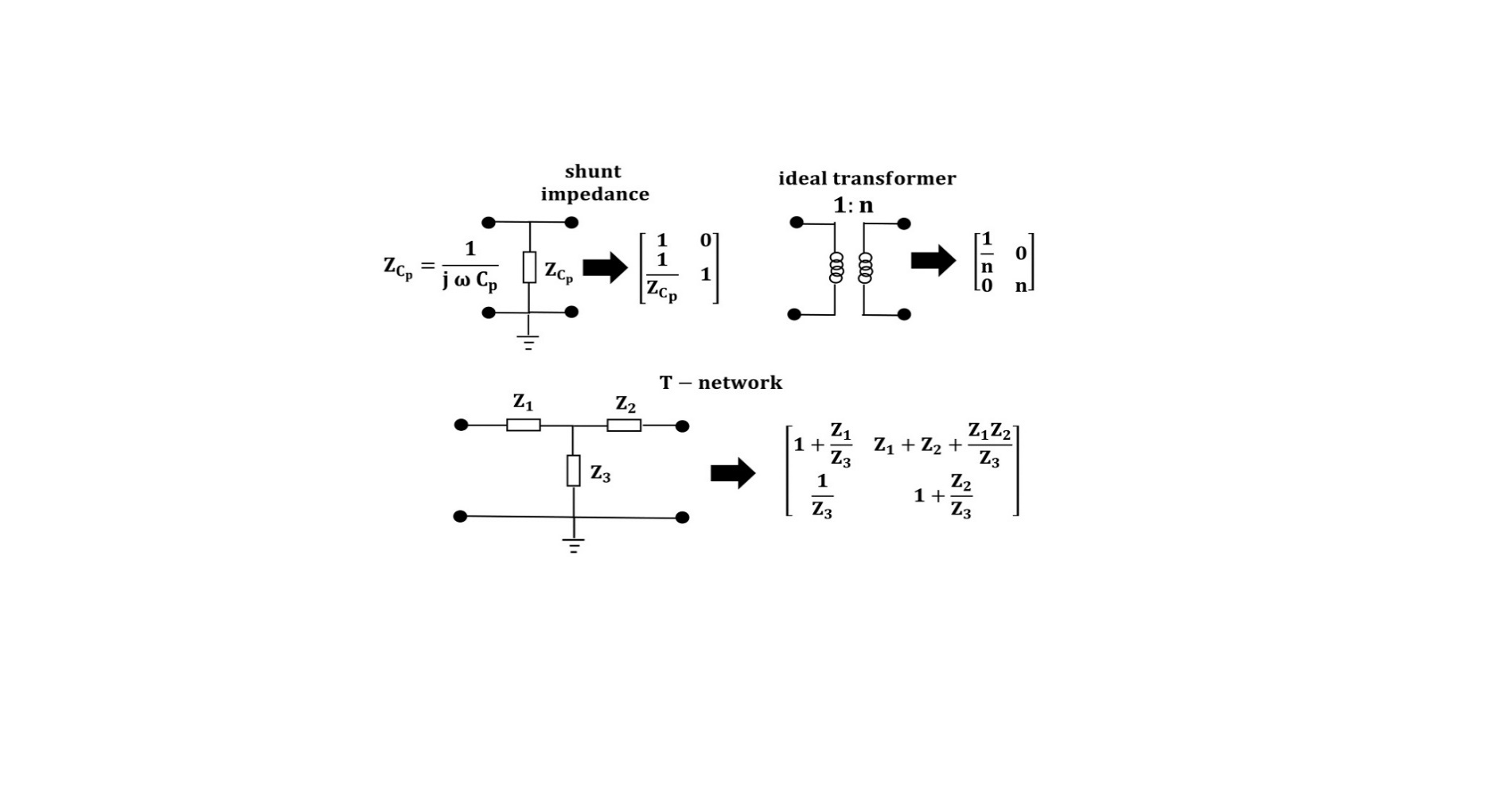** |
| --- |
| **Figure S5.** Instructions to create ABCD matrixes for model of 2-ports Mason’s circuit. |

The resulting ABCD matrix in equation (12S) is the result of the multiplication of all ABCD matrixes representing each single block of the subsystem groups shown in Figure S5.

Moreover, while modelling a piezo structure, is important to consider the losses, which are piezoelectric, $\tan\theta^{'}$, dielectric, $\tan\delta^{'}$, and elastic $\tan\phi^{'}$, ones. It is possible to relate these losses by considering the assumption of IEEE standard [4]:

|  | $2 tan \theta^{'}- \tan\delta^{'}-\tan\phi^{'}=0$ | (13S) |
| --- | --- | --- |

The value of $\tan\delta^{'}$ is usually given by datasheet, while $\tan\phi^{'}$ can be easily calculated as the inverse of the quality factor, $Q$, as: $\tan\phi^{'}=1/Q$, [5]. Finally, $\tan\theta^{'}$ is given by equation (13S).

To introduce the elastic losses of mechanical layers, such as back and front sides of Langevin, is possible to assume these layers as underdamped harmonic oscillators where the angular frequency of oscillation, $\omega_{m}$, and the mechanical quality factor, $Q_{m}$, are given by following equations:

|  | $\omega_{m}=\sqrt{\frac{K}{M}}$ | (14S) |
| --- | --- | --- |
|  | $Q_{m}=\omega_{m}\left( \frac{M}{B} \right)$ | (15S) |

where $K$ is the material stiffness, $M$ is the mass, and $B$ is the damping coefficient that can be defined through the definition of the damping ratio value, $\xi$, as it follows:

|  | $B=\xi B_{C}$ | (16S) |
| --- | --- | --- |

The value of $\xi$ can be found in [6]. The$B_{C}$ parameter is the critical damping coefficient, defined as it follows:

|  | $B_{C}=\sqrt{4 MK}=2M\omega_{m}$ | (17S) |
| --- | --- | --- |

To simulate the losses in materials like steel and aluminum, it is necessary to define the mass $M$ and the stiffness $K$ of materials. Particularly, it is possible to define them as:

|  | $\left\{ \begin{aligned} M= \rho A t \\ K=Y^{E}\left( \frac{A}{t} \right) \end{aligned} \right.$ | (18S) |
| --- | --- | --- |

where $\rho$ is the density, $A$ the cross-section area, $t$ the thickness and $Y^{E}$ the Young’s modulus of mechanical layers. By summarizing, is possible to find elastic losses of mechanical layers by combining the above equations in the following way:

|  | $Q_{m}=\omega_{m}\left( \frac{M}{B} \right)=\frac{1}{{\tan\phi}^{'}}$ | (19S) |
| --- | --- | --- |

Once all the loss values are obtained, they must be inserted in equations (2S)–(5S) and (8S)–(11S) through the definition of lossy piezo equivalent capacitance, $C_{p}^{*}$, lossy turns ratio value of transformer, $n^{*}$, lossy speed of sound, $c^{*}$, and lossy wave number, $k_{e}^{*}$:

|  | $C_{p}^{*}=C_{p}(1-j\tan\delta^{'})$ | (20S) |
| --- | --- | --- |
|  | $n^{*}=n[1-j({\tan\theta}^{'}-{\tan\phi}^{'})]$ | (21S) |
|  | $c^{*}=c\left( 1+j \frac{{\tan\phi}^{'}}{2} \right)$ | (22S) |
|  | $k_{e}^{*}=\frac{\omega}{c^{*}}=k_{e}\left( 1-j \frac{{\tan\phi}^{'}}{2} \right)$ | (23S) |

About the medium layers of the UPD system, once ultrasound wave has been generated by the TX, it propagates through media which are the PDMS layer and the thin SST layer to the air-cavity. A lumped-parameters model shown in Figure S6 describes the propagation through these media.

| 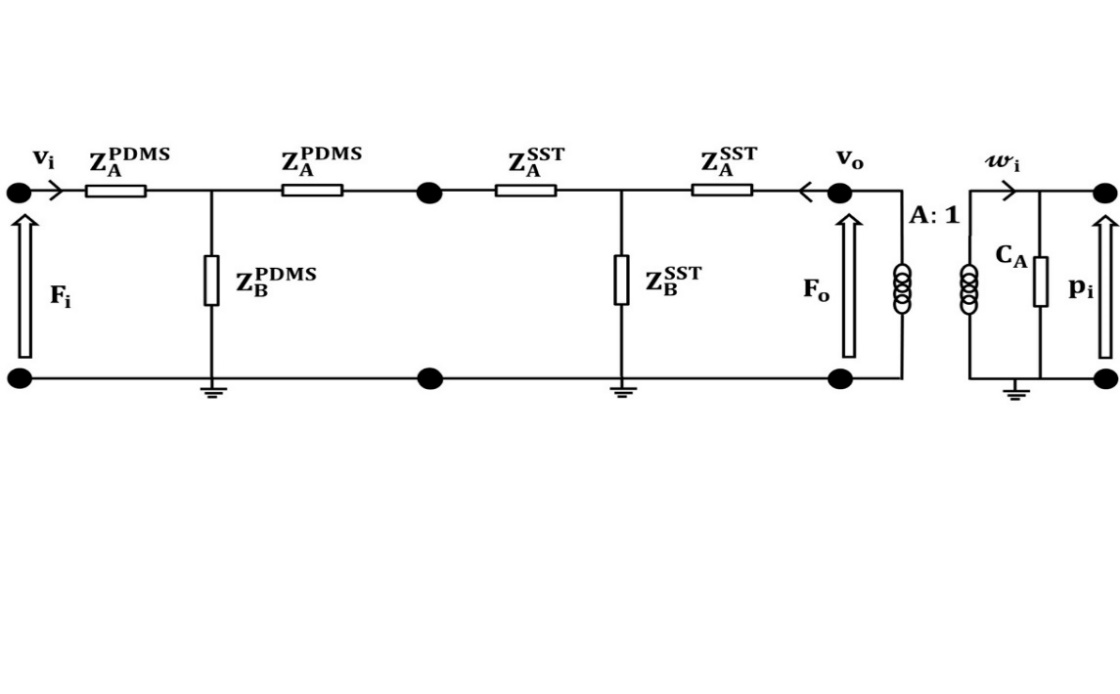 |
| --- |
| **Figure S6.** Lumped parameter model for the wave propagation in the medium layers. |

In Figure S6, the mechanical layers are represented by two T-networks, while the air-cavity is represented by an acoustic compliance, $C_{A}$, which is given by the following equation:

|  | $C_{A}=\frac{\mathrm{Volum}e_{\mathrm{CAVITY}}}{c_{\mathrm{AIR}}^{2} \rho_{\mathrm{AIR}}}$ | (24S) |
| --- | --- | --- |

The mechanical and the acoustic part of the model in Figure S6 are interconnected by the transformer respecting these two domains by the transformer ratio $A$. In the first approximation the value of $A$ corresponds to the area of the steel housing separating the transmission medium and the air cavity.

In Figure 6S, the values of $Z_{A}^{\mathrm{PDMS}}$, $Z_{B}^{\mathrm{PDMS}}$, $Z_{A}^{\mathrm{SST}}$, $Z_{B}^{\mathrm{SST}}$ can be calculated by using equations (2S) and (3S); terms $F_{i}$ and $v_{i}$ are the input force and the velocity at the input side of the PDMS layer, while $F_{o}$ and $v_{o}$ are the output force and the velocity at SST layer side in contact with the air cavity. Terms $\mathcal{w}_{i}$ and $p_{i}$ are the flow rate (volume velocity) and acoustic pressure in the air cavity, which will generate the mechanical vibration of the pMUT diaphragm.

The following Figure S7 shows the structure and the dimensions of the pMUT used in our work as a receiver, and its laminate section. The illustrated pMUT is a laminate structure formed on a silicon (Si) substrate, by a silicon oxide (SiO_2_) thin layer, an aluminum nitride (AlN) piezoelectric thin film covered by Al/Cr metal pads and backed by an air-cavity of cross-section area, $A_{m}$. Electrodes of the pMUT are placed at the edges of the diaphragm above the AlN film. The top electrode is an Al/Cr metal pad while the bottom is formed by a doped silicon layer.

| 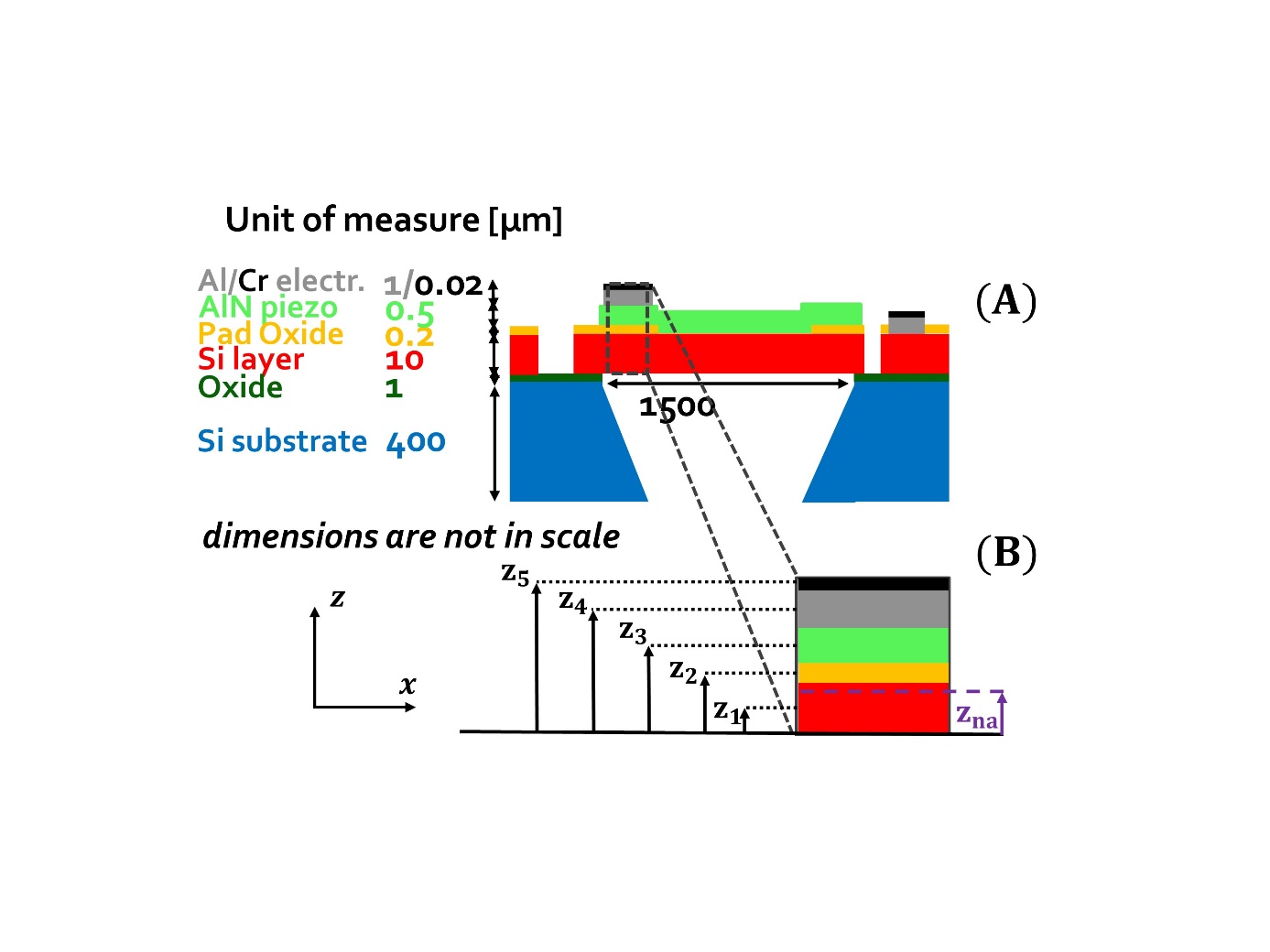 |
| --- |
| **Figure S7.** Square pMUT: structure and dimensions (A); laminate section (B). |

To find the pMUT properties is necessary to define the plate modulus, $Y_{N}$, he location of mid-plane, $z_{N}$, and top, $h_{N}$, of all “$N$” layers constituting the pMUT. Then, it is possible to calculate the location of neutral axis, $z_{\mathrm{na}}$, and the mass per unit area, $\mu$, in order to find the laminate’s flexural rigidity, $D$, of the pMUT [7].

|  | $Y_{N}=\frac{E_{N}}{1-\upsilon^{2}}$ | (25S) |
| --- | --- | --- |
|  | $z_{N}=\sum_{j=1}^{N-1} t_{j}+\frac{1}{2}t_{N}$ | (26S) |
|  | $h_{N}=\sum_{j=1}^{N-1} t_{j}+t_{N}$ | (27S) |
|  | $z_{\mathrm{na}}=\frac{\sum_{N=1}^{N} t_{N}z_{N}Y_{N}}{\sum_{N=1}^{N} t_{N}Y_{N}}$ | (28S) |
|  | $\mu=\sum_{N=1}^{N} t_{N}\rho_{N}$ | (29S) |
|  | $D=\frac{1}{3}\sum_{N=1}^{N} Y_{N}\left( \bar{h}_{N}^{3}-\bar{h}_{N-1}^{3} \right)$ | (30S) |

where $\bar{h}_{N}=h_{N}-z_{\mathrm{na}}$.

In order to find resulting pMUT values for Young’s modulus, $E$, plate modulus, $Y$, Poisson’s ratio, $\upsilon$, and density, $\rho$, parameters, an homogenization technique was adopted:

|  | $E_{\mathrm{pMUT}} =\frac{E_{\mathrm{Si}}S_{\mathrm{Si}}+E_{\mathrm{SiO}_{2}}S_{\mathrm{SiO}_{2}}+E_{\mathrm{AlN}}S_{\mathrm{AlN}}+E_{\mathrm{Al}}S_{\mathrm{Al}}+E_{\mathrm{Cr}}S_{\mathrm{Cr}}}{S_{\mathrm{Si}}+S_{\mathrm{SiO}_{2}}{+S}_{\mathrm{AlN}}{+S}_{\mathrm{Al}}+S_{\mathrm{Cr}}}$ | (31S) |
| --- | --- | --- |
|  | $Y_{\mathrm{pMUT}} =\frac{Y_{\mathrm{Si}}S_{\mathrm{Si}}+Y_{\mathrm{SiO}_{2}}S_{\mathrm{SiO}_{2}}+Y_{\mathrm{AlN}}S_{\mathrm{AlN}}+Y_{\mathrm{Al}}S_{\mathrm{Al}}+Y_{\mathrm{Cr}}S_{\mathrm{Cr}}}{S_{\mathrm{Si}}+S_{\mathrm{SiO}_{2}}{+S}_{\mathrm{AlN}}{+S}_{\mathrm{Al}}+S_{\mathrm{Cr}}}$ | (32S) |
|  | $Y_{\mathrm{pMUT}}=\frac{E_{\mathrm{pMUT}}}{1-\upsilon^{2}} \Leftrightarrow\upsilon=\sqrt{\frac{Y_{\mathrm{pMUT}}-E_{\mathrm{pMUT}}}{Y_{\mathrm{pMUT}}}}$ | (33S) |
|  | $\rho_{\mathrm{pMUT}} =\frac{\rho_{\mathrm{Si}}V_{\mathrm{Si}}+\rho_{\mathrm{SiO}_{2}}V_{\mathrm{SiO}_{2}}+\rho_{\mathrm{AlN}}V_{\mathrm{AlN}}+\rho_{\mathrm{Al}}V_{\mathrm{Al}}+\rho_{\mathrm{Cr}}V_{\mathrm{Cr}}}{V_{\mathrm{Si}}+V_{\mathrm{SiO}_{2}}+V_{\mathrm{AlN}}+V_{\mathrm{Al}}+V_{\mathrm{Cr}}}$ | (34S) |

where $S$ is the cross-section area and $V$ the volume of the layers constituting the pMUT.

Figure S8 shows the pMUT lumped-parameter model divided in electrical, mechanical and acoustic domain. In the mechanical domain, $k^{-1}$ is the compliance, $m_{m}$ the mass and $b_{m}$ the damping of the system.

| **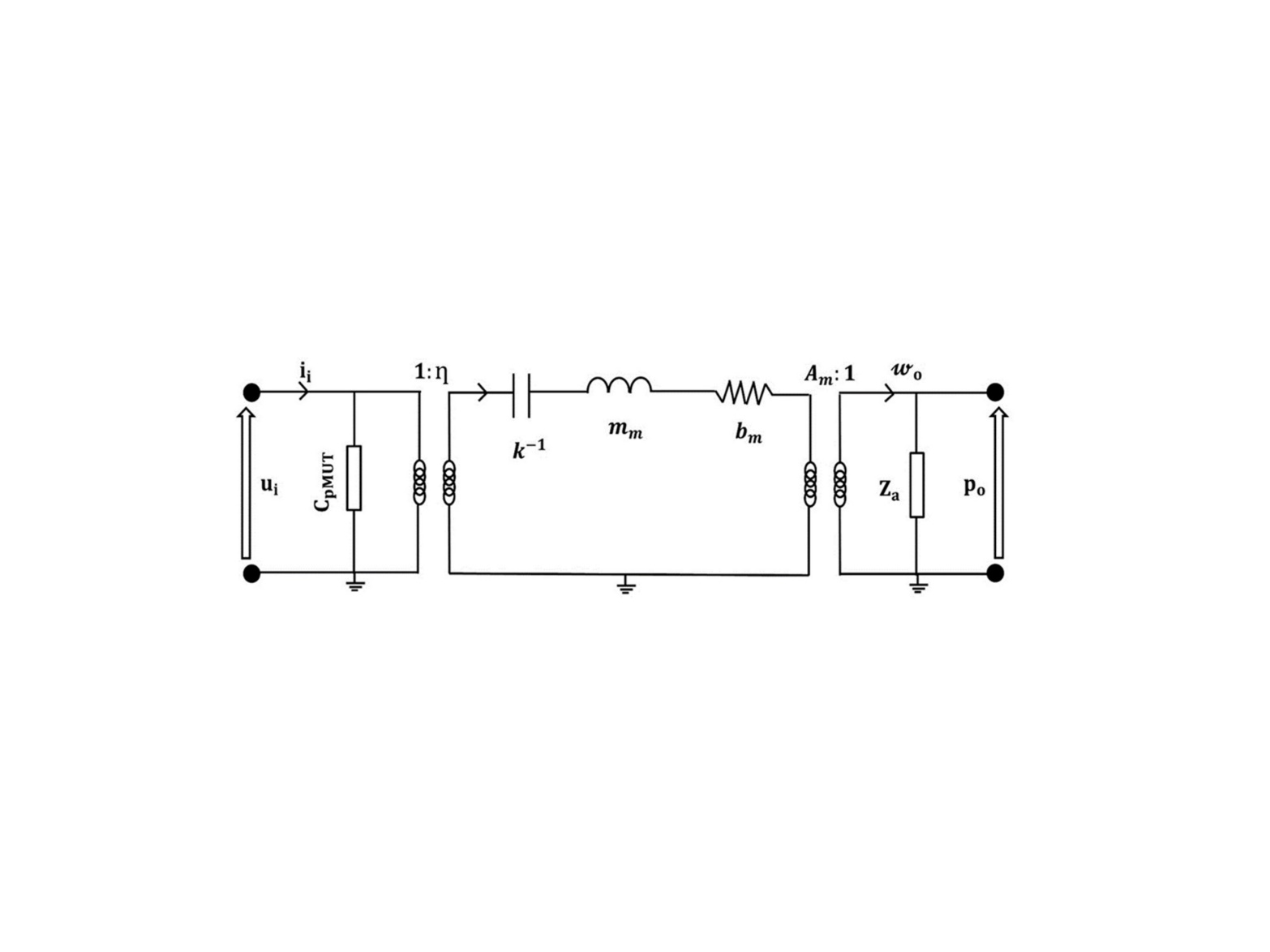** |
| --- |
| **Figure S8.** Square pMUT lumped parameter model. |

The resulting natural angular frequency for the pMUT is given by the following equation [7]:

|  | $\omega=\frac{\lambda^{2}}{l^{2}}\sqrt{\frac{D}{\mu}}=\sqrt{\frac{1}{k^{-1} m_{m}}}$ | (35S) |
| --- | --- | --- |

The value of $\omega$ can be easily deducted from the impedance measurement by means of an impedance analyzer. The impedance measurement, $Z_{\mathrm{pMUT}}$, gives precise information about the values of the resonant frequency, $f_{R}$, and antiresonant frequency, $f_{\mathrm{AR}}$. Moreover, from the impedance measurement, it is possible to calculate the pMUT equivalent capacitance, $C_{\mathrm{pMUT}}$, and the coupling factor, $k_{\mathrm{pMUT}}$.

|  | $C_{\mathrm{pMUT}}=\frac{1}{2\pi f_{R}Z_{\mathrm{pMUT}}}$ | (36S) |
| --- | --- | --- |
|  | $k_{\mathrm{pMUT}}=\sqrt{\left[ 1-\frac{\tan\left( \frac{\pi}{2}\frac{f_{\mathrm{AR}}}{f_{R}} \right)}{\frac{\pi}{2}\frac{f_{\mathrm{AR}}}{f_{R}}} \right]^{-1}}$ | (37S) |

The loss values of pMUT can be calculated from equation (13S). Particularly, $\tan\delta^{'}$ is the value of AlN dielectric loss, while to determine the value of $Q_{\mathrm{pMUT}}$ to get $\tan\phi^{'}$ is possible to proceed experimentally through ring-down methodology.

Once $\omega$ is calculated from the $f_{R}$ value, given by the $Z_{\mathrm{pMUT}}$ measurement, is possible to define the eigenvalue $\lambda^{2}$, as it follows:

|  | $\lambda^{2}=\omega l^{2} \left( \sqrt{\frac{D}{\mu}} \right)^{-1}$ | (38S) |
| --- | --- | --- |

Then, by considering a square plate, two-dimensional resonator, in [7] authors found the following relationship for the effective stiffness, $k$, value:

|  | $k=\left( 166.4 +47.6 \upsilon\right)\frac{D}{l^{2}}$ | (39S) |
| --- | --- | --- |

While computing the $k$ value in simulation, it is important to introduce its elastic loss factor, because, in the mechanical domain, $k$ represents the behavior of a spring [8]. Once $k$ is given, by combining equations (35S) and (39S), is possible to calculate the value of $m_{m}$, as it follows:

|  | $m_{m}=\frac{\mu l^{2}}{\left( \lambda^{2} \right)^{2}} \left( 166.4 +47.6 \upsilon\right)$ | (40S) |
| --- | --- | --- |

Finally, is possible to determine the values of the effective compliance $k^{-1}$, by using equation (35S), and the effective value of damping, $b_{m}$, in the following way:

|  | $k^{-1}=\frac{1}{\omega^{2}m_{m}}$ | (41S) |
| --- | --- | --- |
|  | $b_{m}=\omega\frac{m_{m}}{Q_{\mathrm{pMUT}}}$ | (42S) |

The value of the electromechanical coupling coefficient, $ƞ$, can be calculated by considering the following relationship [7]:

|  | $ƞ=\sqrt{\frac{-C_{\mathrm{pMUT}}k_{\mathrm{pMUT}}^{2}}{\left( k^{-1}k_{\mathrm{pMUT}}^{2}-k^{-1} \right)}}$ | (43S) |
| --- | --- | --- |

About the acoustic domain of Figure S8, the parameter $Z_{a}$ is the pMUT radiation impedance and it is described, under free-field conditions, as it follows [9]:

|  | $Z_{a}=R_{a}+{j\omega L}_{a}$ | (44S) |
| --- | --- | --- |

In order to estimate the value of the air acoustic impedance for the clamped diaphragm, $\left| Z_{a} \right|$, it is possible to use the Bouwkamp’s formula based on values of wave number, $k_{e}$, and $a$ parameters, in order to get the normalized acoustic impedance [10]. For the proposed pMUT: $2k_{e}a=$ $2k_{e}a=$ 1.84. So, to obtain the air acoustic impedance, we multiplied the normalized acoustic impedance by $\frac{\rho_{\mathrm{air}}c_{\mathrm{air}}}{S}$:

|  | $\left\vert Z_{a} \right\vert=\sqrt{R_{a}^{2}+\omega^{2}L_{a}^{2}}= 1.84 \frac{\rho_{\mathrm{air}}c_{\mathrm{air}}}{S}$ | (45S) |
| --- | --- | --- |

In order to find the value of ${\omega L}_{a}$, it was used the following approximation [10] for estimating the resonant frequency in air:

|  | $f_{R}=\frac{1}{2\pi\sqrt{\left( m_{m}+A_{m}^{2} L_{a} \right)k^{-1}}} \Leftrightarrow A_{m}^{2} L_{a}=\frac{1}{k^{-1}}\left( \frac{1}{4\pi^{2}f_{R}^{2}}-m_{m}k^{-1} \right)$ | (46S) |
| --- | --- | --- |
|  | $\omega L_{a}=\frac{1}{k^{-1}}\left( \frac{1}{4\pi^{2}f_{R}^{2}}-m_{m}k^{-1} \right)\frac{2\pi f_{R}}{\left( \frac{S}{3} \right)^{2}}$ | (47S) |

Where the cross-section area of pMUT back-air cavity, $A_{m}$, can be approximated to a third of AlN diaphragm cross-section area, $S$, [11]. Then, the value of $R_{a}$ is calculated starting from equation (45S).

The following Table 1S shows the parameter values for the screw bolt, back side and front side of Langevin (TX), and Table 2S shows the parameter values of the medium layers.

**Table 1.** S. Parameter values of screw bolt, back side and front side of Langevin (TX).

| **Parameter** | **Screw bolt**  **(steel)** | **Back side**  **(steel)** | **Front side**  **(aluminum)** |
| --- | --- | --- | --- |
| Cross-section Area $\left( S \right)$ [m^2^] | 5.0265·10^-05^ | 9.1185·10^-04^ | 9.62·10^-04^ , 1.6·10^-03^ |
| Thickness $\left( t \right)$ [m] | 0.01545 + 0.0115 | 0.01545 | 0.0268 |
| Density $\left( \rho\right)$ [kg/m^3^] | 7800 | 7800 | 2700 |
| Young's Modulus $(E)$ [Pa] | 2.09·10^+11^ | 2.09·10^+11^ | 1.1·10^+11^ |
| Acoustic Impedance [MRayls] | 40.2 | 40.2 | 17.2 |
| Damping ratio ($\xi$) | 7/1000 | 7/1000 | 2/1000 |

**Table 2.** S. Parameter values of medium layers.

| **Parameter** | **PDMS** | **Tipe 316L SST** | **Air cavity** |
| --- | --- | --- | --- |
| Cross-section Area $\left( S \right)$ [m^2^] | 1.023·10^-03^ | 9.6·10^-04^ | 1·10^-04^ |
| Thickness $\left( t \right)$ [m] | 7·10^-03^ | 3.7·10^-04^ | 1.25·10^-03^ |
| Density $\left( \rho\right)$ [kg/m^3^] | 1063 | 8000 | 1.225 |
| Young's Modulus $(E)$ [N/m^2^] | 1.0076·10^+09^ | 2.09·10^+11^ | - |
| Acoustic Impedance [MRayls] | 1.03 | 40.2 | - |
| Damping ratio ($\xi$) | 7/10 | 7/1000 | - |

References:

1. Zhang, J.-G.; Long, Z.-L.; Ma, W.-J.; Hu, G.-H.; Li, Y.-M. Electromechanical dynamics model of ultrasonic transducer in ultrasonic machining based on equivalent circuit approach. Sensors 2019, 19(6), 1405, DOI: 10.3390/s19061405.
2. Sherrit, S.; Leary, S.P.; Dolgin, B.P.; Bar-Cohen, Y. Comparison of the Mason and KLM equivalent circuits for piezoelectric resonators in the thickness mode. In Proceedings of the IEEE Ultrasonics Symposium, Volume 2, Tahoe, USA, Oct. 1999.
3. Kim, J.; Lee, J. Theoretical Resonance Analysis of Langevin Transducers with Equivalent Circuit Models for Therapeutic Ultrasound. J. Electr. Eng. Technol. 2019, 14(6), 2437–2445, DOI: 10.1007/s42835-019-00275-x.
4. Meeker, T.R. Publication and proposed revision of ANSI/IEEE standard 176-1987 “ANSI/IEEE standard on piezoelectricity”. IEEE Trans. Ultrason. Ferroelectr. Freq. Control 1996, 43(5), 717–771, DOI: 10.1109/TUFFC.1996.535477.
5. Uchino, K. High-Power Piezoelectrics and Loss Mechanisms. In: Advanced Piezoelectric Materials: science and technology, 2nd edition, Publisher: Woodhead Publishing, 2017, pp. 647–754.
6. Ashby, M.F. Material Property Charts. In Materials selection in mechanical design, 4th ed.; Publisher: Elsevier Ltd., UK, 2011; pp. 57–96.
7. Horsley, D.; Lu, Y.; Rozen, O. Flexural Piezoelectric Resonators. In Piezoelectric MEMS Resonators, Bhurgra, H.; Piazza, G. Eds.; Publisher: Springer International Publishing AG, 2017, pp. 153–173.
8. Marzencki, M.; Basrour, S. Modeling of Piezoelectric MEMS Vibration Energy Harvesters. In MEMS: Fundamental Technology and Applications, Choudhary, V.; Iniewski, K., Eds.; Publisher: CRC Press, 2013, pp. 131–160.
9. Przybyla, R.J.; Shelton, S.E.; Guedes, A.; Izyumin, I.I.; Kline, M.H.; Horsley, D.A.; Boser, B.E. In-Air Rangefinding With an AlN Piezoelectric Micromachined Ultrasound Transducer. IEEE Sens. J. 2011, 11(11), 2690–2697, DOI: 10.1109/JSEN.2011.2157490.
10. Halbach, A.; Gijsenbergh, P.; Jeong, Y.; Billen, M.; Chare, C.; Gao, H.; Torri, G.B.; Cheyns, D.; Rottenberg, X.; Rochus V. Modelling of display-compatible piezoelectric micromachined ultrasonic transducers for haptic feedback. In Proceedings of 20th International Conference on Thermal, Mechanical and Multi-Physics Simulation and Experiments in Microelectronics and Microsystems (EUROSIME), Hannover, Germany, Mar. 2019, DOI: 10.1109/EuroSimE.2019.8724526.
11. Horsley, D.A.; Rozen, O.; Lu, Y.; Shelton, S.; Guedes, A.; Przybyla, R.; Tang, H.-Y.; Boser, B.E. Piezoelectric Micromachined Ultrasonic Transducers for Human-Machine Interfaces and Biometric Sensing. In Proceedings of the IEEE Sensors Conference, Busan, South Korea, Nov. 2015, DOI: 10.1109/ICSENS.2015.7370564.
